# Adipocyte-derived extracellular vesicles regulate survival and function of pancreatic **β** cells

**DOI:** 10.1172/jci.insight.141962

**Published:** 2021-03-08

**Authors:** Iacopo Gesmundo, Barbara Pardini, Eleonora Gargantini, Giacomo Gamba, Giovanni Birolo, Alessandro Fanciulli, Dana Banfi, Noemi Congiusta, Enrica Favaro, Maria Chiara Deregibus, Gabriele Togliatto, Gaia Zocaro, Maria Felice Brizzi, Raul M. Luque, Justo P. Castaño, Maria Alessandra Bocchiotti, Simone Arolfo, Stefania Bruno, Rita Nano, Mario Morino, Lorenzo Piemonti, Huy Ong, Giuseppe Matullo, Juan M. Falcón-Pérez, Ezio Ghigo, Giovanni Camussi, Riccarda Granata

**Affiliations:** 1 Division of Endocrinology, Diabetes and Metabolism, and; 2 Department of Medical Sciences, University of Turin, Turin, Italy.; 3 Italian Institute for Genomic Medicine, Turin, Italy.; 4 Candiolo Cancer Institute, FPO Istituto di Ricovero e Cura a Carattere Scientifico, Candiolo, Italy.; 5 2i3T Business Incubator and Technology Transfer and; 6 Molecular Biotechnology Center, University of Turin, Turin, Italy.; 7 Maimonides Institute for Biomedical Research of Córdoba, Department of Cell Biology, Physiology and Immunology, University of Córdoba, and Reina Sofia University Hospital, Córdoba, Spain.; 8 Department of Reconstructive and Aesthetic Plastic Surgery and; 9 Department of Surgical Sciences, University of Turin, Turin, Italy.; 10 Diabetes Research Institute, IRCCS San Raffaele Scientific Institute, and Vita-Salute San Raffaele University, Milan, Italy.; 11 Faculty of Pharmacy, University of Montréal, Montréal, Québec, Canada.; 12 Exosomes Laboratory and; 13 Metabolomics Platform, CIC bioGUNE, Bizkaia Technology Park, Derio, Spain.; 14 Centro de Investigación Biomédica en Red de Enfermedades Hepáticas y Digestivas, Madrid, Spain.; 15 Ikerbasque, Basque Foundation for Science, Bilbao, Bizkaia, Spain.

**Keywords:** Metabolism, Adipose tissue, Beta cells, Diabetes

## Abstract

Extracellular vesicles (EVs) are implicated in the crosstalk between adipocytes and other metabolic organs, and an altered biological cargo has been observed in EVs from human obese adipose tissue (AT). Yet, the role of adipocyte-derived EVs in pancreatic β cells remains to be determined. Here, we explored the effects of EVs released from adipocytes isolated from both rodents and humans and human AT explants on survival and function of pancreatic β cells and human pancreatic islets. EVs from healthy 3T3-L1 adipocytes increased survival and proliferation and promoted insulin secretion in INS-1E β cells and human pancreatic islets, both those untreated or exposed to cytokines or glucolipotoxicity, whereas EVs from inflamed adipocytes caused β cell death and dysfunction. Human lean adipocyte-derived EVs produced similar beneficial effects, whereas EVs from obese AT explants were harmful for human EndoC-βH3 β cells. We observed differential expression of miRNAs in EVs from healthy and inflamed adipocytes, as well as alteration in signaling pathways and expression of β cell genes, adipokines, and cytokines in recipient β cells. These in vitro results suggest that, depending on the physiopathological state of AT, adipocyte-derived EVs may influence β cell fate and function.

## Graphical abstract

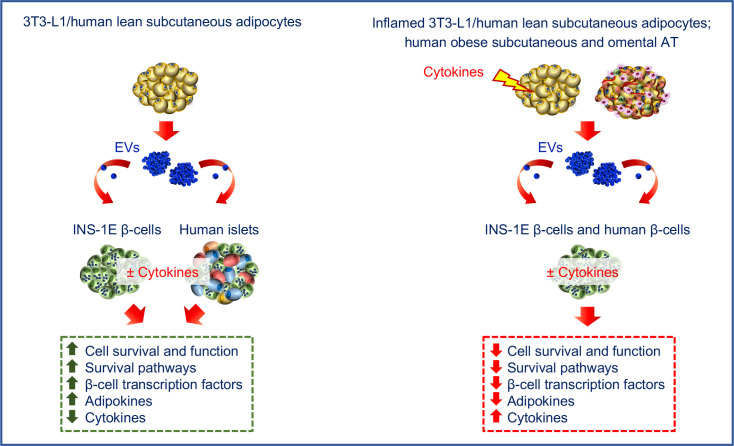

## Introduction

Obesity is a complex multifactorial disease, with an incidence that has reached epidemic proportions. Features of obesity-induced metabolic and inflammatory diseases include accumulation of ectopic fat in key insulin-sensitive organs, inflammation of white adipose tissue (AT), and infiltration of proinflammatory macrophages, all contributing to the development of insulin resistance, type 2 diabetes (T2D), cardiovascular and neurodegenerative diseases, and cancer (1).

AT is a complex and dynamic endocrine organ and a master regulator of whole-body homeostasis through release of adipokines, lipids, metabolites, noncoding RNAs, and extracellular vesicles (EVs), which act on metabolic tissues to regulate lipid and glucose homeostasis. However, whereas healthy AT has a positive systemic effect, the secretion of adipocyte-specific factors is often dysregulated with obesity, contributing to low-grade chronic inflammation and insulin resistance (1–3). Indeed, AT-derived inflammatory cytokines (CKs), as well as circulating free fatty acids (FFA) and high levels of glucose, all cause pancreatic β cell apoptosis and contribute to the loss of β cell mass and function, characteristics of T2D (4, 5). Moreover, behavioral differences exist among AT depots, as visceral fat has been associated with metabolic dysfunction, while subcutaneous AT (SAT) has been associated with beneficial effects on metabolism (1, 6).

EVs are membrane-bound particles released from all types of cells and important mediators of intercellular communication among different organs and tissues. They deliver proteins, mRNAs, and noncoding RNAs, such as miRNAs, to recipient cells, thereby modulating their phenotype. EVs are involved in many physiological processes and in the pathogenesis of different diseases, including metabolic and neurodegenerative diseases and cancer (7). Moreover, the nature of their bioactive cargo is heavily dependent on the cell of origin, its physiopathological state, and the stimulus for release (8, 9).

EVs and their components have been identified as potential diagnostic and prognostic biomarkers in metabolic diseases. Indeed, EVs from in vitro human differentiated adipocytes and SAT and omental AT (OAT) explants express different adipocyte-specific proteins and immune factors (8, 10–12). Moreover, altered miRNA profiles have been described in EVs from patients with obesity and metabolic syndrome (8, 13, 14).

EVs isolated from SAT and OAT were found to promote the differentiation of monocytes to inflammatory macrophages and to reduce insulin signaling in hepatocytes and muscle cells (10, 11). Moreover, EVs from AT of *ob/ob* mice induced macrophage infiltration into AT and liver and caused insulin resistance (15). Similarly, EVs from AT macrophages of obese mice induced insulin resistance, whereas those from lean mice increased insulin sensitivity in obese mice (16). Furthermore, while EVs from adipose-derived stem cells of lean individuals or from mice fed with normal diet exhibited regenerative properties and beneficial metabolic effects, EVs from individuals with obesity and T2D showed reduced therapeutic potential (17–19). Overall, these findings suggest that both AT depot and the physiopathological state of the donor influence EV cargo and its effects in recipient cells.

The content of EVs released by rat and human pancreatic islets and β cells in both normal and stress conditions and their effects in recipient cells, including β cells, islet endothelial cells, and immune cells, have been recently studied (20–22). The EV-mediated crosstalk between different cell types and pancreatic islets/β cells has been also reported. Indeed, human T lymphocyte–derived EVs induced apoptosis in β cells and promoted type 1 diabetes (T1D) in mice (23). In addition, EVs from endothelial progenitor cells enhanced neoangiogenesis in human pancreatic islets (24), whereas EVs from insulin-resistant muscles influenced gene expression and proliferation in mouse β cells (25). However, the EV-mediated crosstalk between adipocytes and pancreatic β cells remains unexplored.

In the present study, we describe the effects of EVs isolated from healthy or inflamed rodent and human adipocytes on survival, proliferation, and function of rodent and human β cells and human pancreatic islets, untreated or exposed to diabetogenic stimuli. We also show the role of EVs obtained from human lean and obese AT explants on human β cell fate and function. The survival and metabolic pathways, along with expression of β cell genes, adipokines and CKs, were assessed in EV-treated β cells, as well as the differential expression of miRNAs in EVs. Our findings suggest the existence of an EV-mediated functional crosstalk between AT and pancreatic β cells, which positively or negatively influences β cell fate, depending on the physiopathological state of adipocytes and AT of origin.

## Results

### EVs isolated from 3T3-L1 adipocytes and human SAT transport adipocyte-specific genes and proteins.

**Figure 1:**
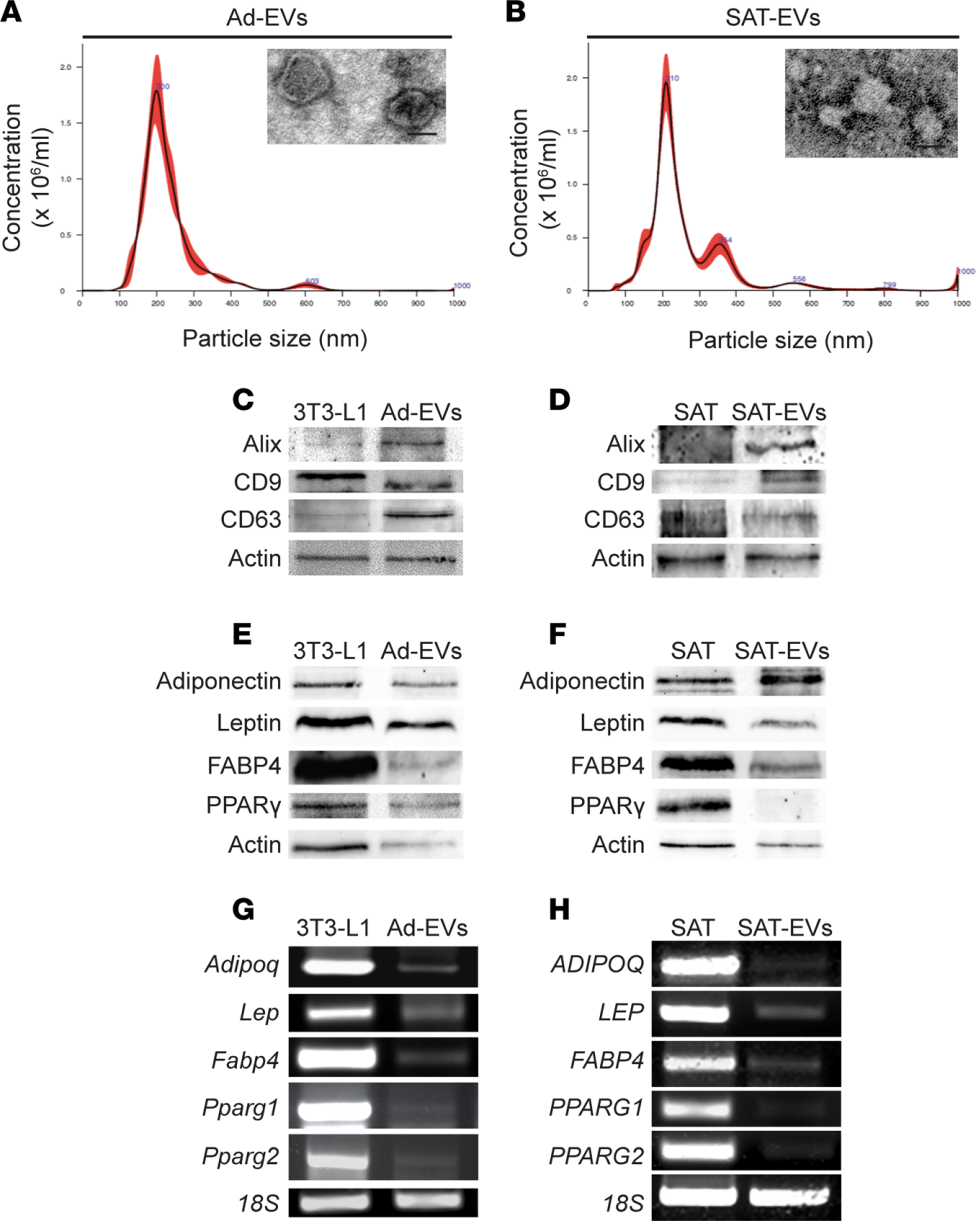
Characterization of EVs released from 3T3-L1 adipocytes and human subcutaneous adipose tissue. Analysis of size distribution, showing the size and number of EVs released from 3T3-L1 adipocytes (Ad-EVs) (**A**) and human subcutaneous adipose tissue (SAT-EVs) (**B**) using the NanoSight technology (*n* = 3). Insets show representative micrographs obtained by TEM of purified Ad-EVs and SAT-EVs (scale bar: 100 nm). (**C** and **D**) Representative Western blot images for EV markers Alix, CD9, and CD63 in 3T3-L1 adipocytes and Ad-EVs (**C**) and in SAT and SAT-EVs (**D**) (*n* = 3). (**E** and **F**) Representative Western blot for adiponectin, leptin, FABP4, and PPARγ in 3T3-L1 cells and Ad-EVs (**E**) and in SAT and SAT-EVs (**F**). Actin served as internal control (*n* = 3). (**G** and **H**) Representative gene expression for adiponectin, leptin, FABP4, PPARγ1, and PPARγ2 in 3T3-L1 and Ad-EVs (**G**) and in SAT and SAT-EVs (**H**), as assessed by RT-PCR. 18s rRNA served as internal control (*n* = 3).

EVs isolated from 3T3-L1 adipocytes (Ad-EVs) and human SAT (SAT-EVs) were isolated by ultracentrifugation from 3T3-L1 adipocytes and human SAT, respectively. To assess their number and size, both types of EVs were first analyzed by nanoparticle tracking analysis (NTA) (Figure 1, A and B). Their round-shape morphology and size were further confirmed by transmission electron microscopy (TEM) (Figure 1, A and B, insets). We observed variability in the size of EVs, depending on whether they were studied by NTA or TEM, likely because of the influence of temperature and Brownian motion for NTA, and fixation of EVs for TEM. Western blot analysis showed expression of the EV markers Alix, CD9, and CD63 in both Ad-EVs and SAT-EVs (Figure 1, C and D). Adipocyte-specific proteins and mRNAs, such as adiponectin, leptin, fatty acid–binding protein 4 (FABP4), and PPARγ, were detected in both 3T3-L1 cells (Figure 1, E and G) and SAT (Figure 1, F and H) and, although in lower amount, also in their respective EVs, except PPARγ, which was absent in SAT-EVs.

### EVs from normal or inflamed 3T3-L1 adipocytes produce opposite effects on survival and function of pancreatic β cells.

**Figure 2:**
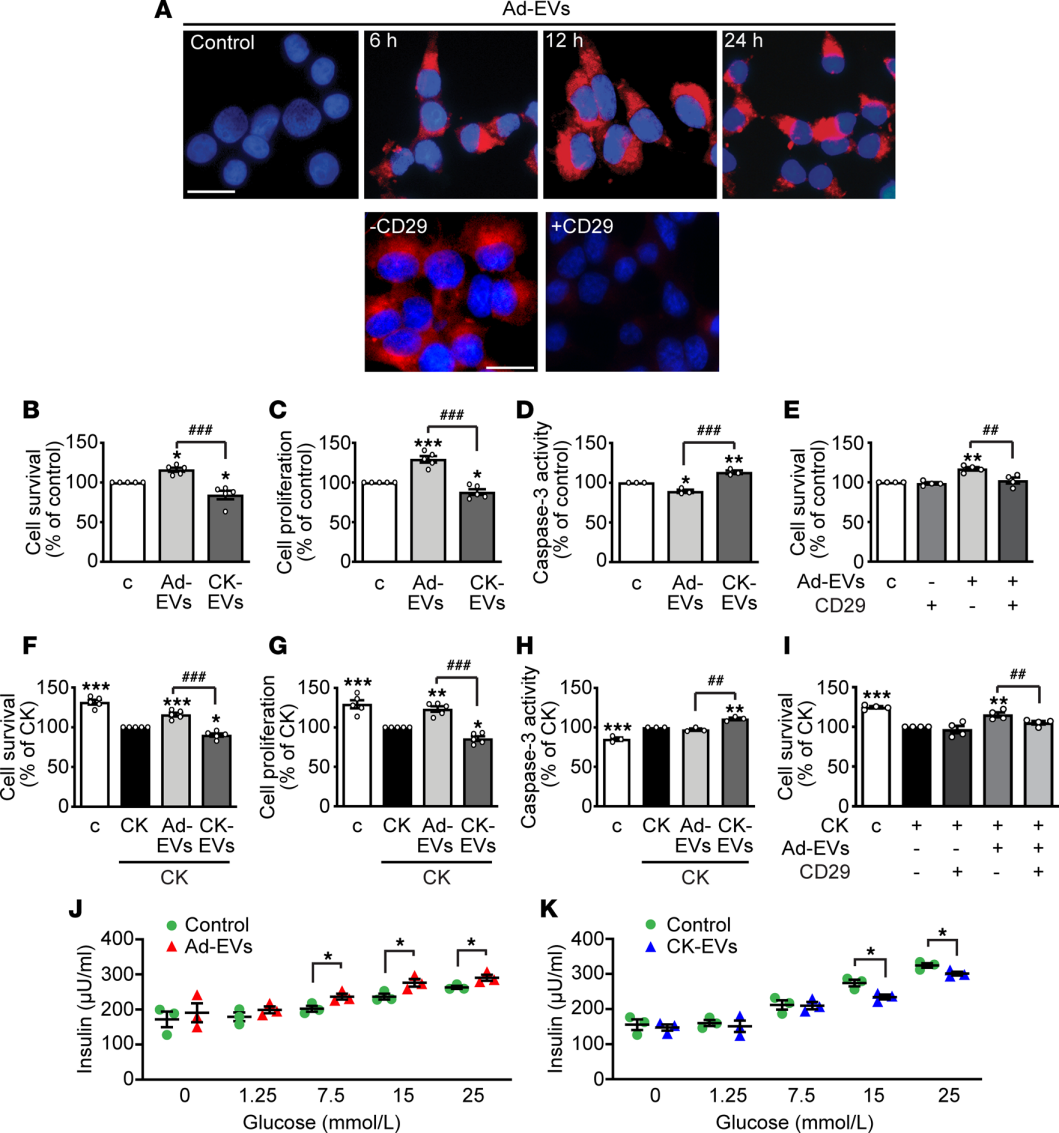
Effect of Ad-EVs and CK-EVs on survival, proliferation, apoptosis, and function of INS-1E β cells. (**A**) Top: Representative fluorescence microscopy micrographs showing internalization of Ad-EVs in INS-1E β cells, incubated at the indicated times with unlabeled Ad-EVs (Control) or with Ad-EVs labeled with PKH26 (red dye) (scale bar: 10 μm). Bottom: Internalization of Ad-EVs for 24 hours in cells untreated (-CD29) or preincubated for 1 hour with the blocking antibody against CD29 (+CD29). Nuclei were stained blue with DAPI (scale bar: 10 μm). Cell survival (**B**), cell proliferation (**C**), and apoptosis (**D**) assessed by MTT, BrdU, and caspase-3 activity, respectively, in cells cultured in serum-deprived medium for 12 hours, then untreated (control [c]) or treated for a further 24 hours with Ad-EVs (10 × 10^3^/cell) or with EVs from 3T3-L1 adipocytes treated for 24 hours with cytokines (CKs) (CK-EVs) (TNF-α/IFN-γ/IL-1β [50, 25, and 2.5 ng/mL, respectively]). (**E**) Cell survival in cells treated for 24 hours with or without Ad-EVs and anti-CD29 antibody. Results are expressed as percentage of control (mean ± SEM). *P < 0.05, **P < 0.01, ***P < 0.001 vs. c; ^##^*P* < 0.01, ^###^*P* < 0.001 by 1-way ANOVA and Tukey’s post hoc test (*n* = 5 for **B** and **C**; *n* = 3 for **D**; *n* = 4 for **E**). Cell survival (**F**), cell proliferation (**G**), and apoptosis (**H**) assessed by MTT, BrdU, and caspase-3 activity, respectively, in β cells cultured in serum-deprived medium or pretreated for 40 minutes with CKs (TNF-α/IFN-γ/IL-1β [100, 50 and 5 ng/mL, respectively]), and then with EVs or CK-EVs for 24 hours. (**I**) Cell survival in cells cultured with or without CKs, Ad-EVs, and CD29 blocking antibody. Results are expressed as mean ± SEM. *P < 0.05, **P < 0.01, ***P < 0.001 vs. CK; ^##^*P* < 0.01, ^###^*P* < 0.001 by 1-way ANOVA and Tukey’s post hoc test (*n* = 5 for **F** and **G**; *n* = 3 for **H**; *n* = 4 for **I**). (**J** and **K**) Insulin secretion assessed by ELISA in INS-1E β cells incubated with 2 mM glucose for 1 hour and for a further 1 hour with the indicated concentrations of glucose, in the presence or absence of Ad-EVs (**J**) or CK-EVs (**K**) (mean ± SEM). *P < 0.05 by 2-tailed Student’s t test (*n* = 3).

To assess the biological effects of Ad-EVs in β cells, we first explored whether Ad-EVs were efficiently internalized by INS-1E β cells. Ad-EVs labeled with PKH26 dye were incorporated by β cells and accumulated at the perinuclear area after 6, 12, and 24 hours (Figure 2A, top). However, pretreatment with anti-CD29 blocking antibody inhibited internalization (Figure 2A, bottom), suggesting a role for adhesion molecules in Ad-EV uptake by β cells. We next investigated the effect of Ad-EVs on survival and function of β cells. INS-1E β cells were cultured in the absence of serum and challenged with different doses of Ad-EVs. From 10 to 15 × 10^3^/cell, Ad-EVs counteracted serum deprivation–induced cell death, suggesting survival effects (Supplemental Figure 1A; supplemental material available online with this article; https://doi.org/10.1172/jci.insight.141962DS1). Based on these findings, 10 × 10^3^ was selected as the EV concentration for subsequent experiments. The role of Ad-EVs was next studied in β cells cultured in the presence of TNF-α, IL-1β, and IFN-γ, whose synergism has been implicated in β cell death and dysfunction in both T1D and T2D (4, 26, 27). Untreated and CK-treated β cells were challenged with Ad-EVs or EVs from 3T3-L1 adipocytes exposed to the same combination of CKs (CK-EVs). Indeed, elevation of inflammatory CKs in obesity and T2D causes adipocyte dysfunction and insulin resistance in AT, inflammation, and metabolic damage (1, 3). Ad-EVs from untreated adipocytes increased cell survival and proliferation and reduced apoptosis, assessed as caspase-3 activity, in serum-deprived cells (Figure 2, B–D). Similar effects were obtained in CK-treated β cells, except for apoptosis that was not blunted by Ad-EVs, compared with CKs alone (Figure 2, F–H). As opposed to Ad-EVs, CK-EVs reduced cell survival and proliferation and increased apoptosis in serum-starved cells (Figure 2, B–D); furthermore, CK-EVs exacerbated the detrimental effects of CKs in β cells, including an effect on apoptosis (Figure 2, F–H). Of note, pretreatment with anti-CD29 antibody, while having no effect per se, blocked the survival action of Ad-EVs, under both serum starvation and treatment with CKs (Figure 2, E and I). The functions of Ad-EVs and CK-EVs were assessed also in β cells exposed to glucolipotoxicity, which adversely affects β cell function and survival in T2D (4). Ad-EVs increased, while CK-EVs reduced, survival and proliferation of β cells treated with high concentrations of glucose and palmitate (Supplemental Figure 1, B and C). Anti-CD29 antibody attenuated the survival effects of Ad-EVs, in line with the results observed for serum starvation and CK synergism (Supplemental Figure 1D).

Ad-EVs also slightly increased glucose-stimulated insulin secretion (GSIS), at 7.5, 15, and 25 mM glucose (Figure 2J), whereas CK-EVs inhibited GSIS at the highest glucose concentrations tested (15 and 25 mM) (Figure 2K), in agreement with the results on cell survival and proliferation. Overall, these data indicate that EVs derived from healthy adipocytes are beneficial, whereas EVs from inflamed adipocytes are harmful for β cells, and furthermore, internalization by β cells is essential for EVs to display their effects.

### Ad-EVs and CK-EVs modulate survival and unfolded protein response pathways in pancreatic β cells as well as expression of β cell– and adipocyte-specific genes and CKs.

**Figure 3:**
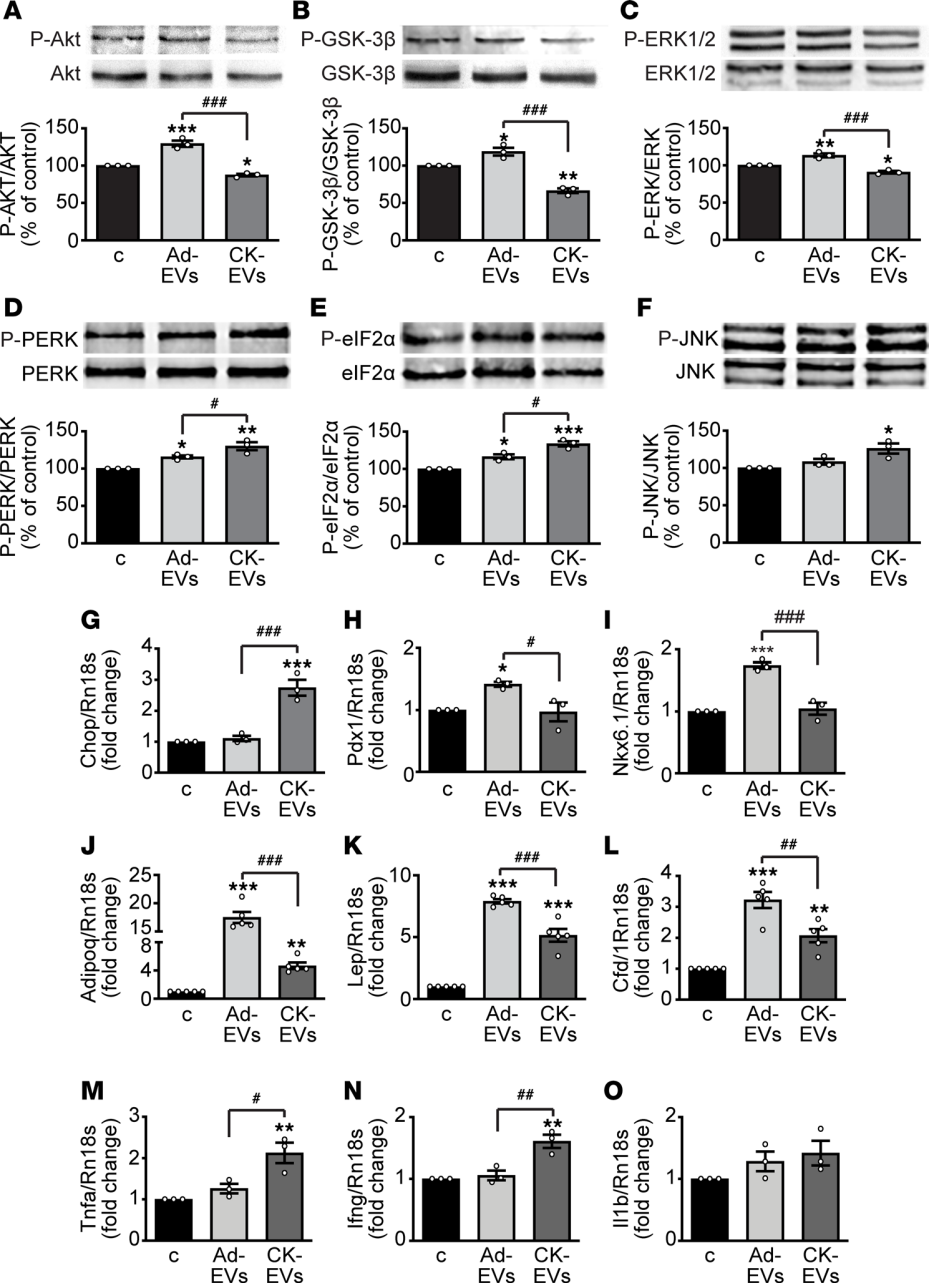
Regulation of signaling pathways and expression of β cell genes, adipokines, and CKs in INS-1E β cells treated with Ad-EVs and CK-EVs. Representative Western blots for phosphorylated Akt (P-Akt) (**A**), P-GSK-3β (**B**), P-ERK1/2 (**C**), P-PERK (**D**), P-eIF2α (**E**), and P-JNK (**F**) in cells untreated (control [c]) or treated with Ad-EVs or CK-EVs for 24 hours (top). Blots were reprobed with nonphosphorylated antibodies for normalization (bottom). Results are expressed as a percentage of control untreated cells (mean ± SEM). *P < 0.05, **P < 0.01, ***P < 0.001 vs. c; ^#^*P* < 0.05, ^###^*P* < 0.001 by 1-way ANOVA and Tukey’s post hoc test (*n* = 3). Real-time PCR for the UPR gene Chop (**G**) and for β cell genes, *Pdx1* (**H**) and *Nkx6.1* (**I**); adipokines: adiponectin (*Adipoq*) (**J**), leptin (*Lep*) (**K**), and adipsin (*Cfd*, complement factor D) (**L**); and CKs: TNF-α (*Tnfa*) (**M**), IFN-γ (*Ifng*) (**N**), and IL-1β (*Il1b*) (**O**) in β cells untreated or treated with Ad-EVs or CK-EVs for 24 hours (mean ± SEM). *P < 0.05, **P < 0.01, ***P < 0.001 vs. c; ^#^*P* < 0.05, ^##^*P* < 0.01, *^###^P* < 0.001 by 1-way ANOVA and Tukey’s post hoc test (*n* = 3 for **G**–**I** and **M**–**O**; *n* = 5 for **J**–**L**).

The signaling pathways involved in the effects of Ad-EVs and CK-EVs in β cells were next studied. Treatment of INS-1E β cells for 24 hours with Ad-EVs increased, whereas CK-EVs reduced, the phosphorylation of Akt (Figure 3A), its downstream target GSK-3β (Figure 3B), and MAPK ERK1/2 (Figure 3C), key pathways regulating survival, proliferation, and function of β cells (28). We then analyzed the effects of EVs on the unfolded protein response (UPR), an adaptive response of the cells to the disruption of endoplasmic reticulum (ER) homeostasis. In β cells the UPR, which is activated by elevated levels of FFAs/glucose or CKs, among others, can restore protein folding, cell function, and survival; however, when unable to compensate, it ultimately leads to apoptotic cell death (29, 30). Ad-EVs, and to a greater extent CK-EVs, increased the phosphorylation of PKR-like ER kinase (PERK) and its downstream effector, eukaryotic initiation factor 2α (eIF2α) (Figure 3, D and E). Furthermore, CK-EVs but not Ad-EVs, promoted the phosphorylation of c-Jun N-terminal kinase (JNK) (Figure 3F), which can be activated by the UPR component inositol-requiring protein 1α (IRE1α). Importantly, only CK-EVs were able to increase the mRNA levels of the proapoptotic transcription factor C/EBP homologous protein (CHOP) (Figure 3G), whose activity is promoted by both PERK/eIF2α and IRE1α/JNK (31).

With regards to the regulation of β cell genes, Ad-EVs and CK-EVs, respectively, promoted and blunted the expression of transcription factors essential for β cell differentiation/maturation and β cell function and identity, such as pancreatic and duodenal homeobox 1 (*Pdx1*) and *Nkx6.1* (32) (Figure 3, H and I). We then assessed the presence of adipocyte-specific mRNAs in INS-1E β cells, such as *Adipoq*, encoding for adiponectin, which positively modulates β cell survival (2); *Lep*, whose product, leptin, has antiapoptotic effects on β cells but inhibits GSIS (2); and complement factor D (*Cfd*), encoding for adipsin, which stimulates GSIS (33). Adiponectin gene expression was strongly increased in cells exposed to Ad-EVs; expression of leptin and adipsin was also increased, though to a lesser extent, also increased the mRNA levels of these adipokines (Figure 3, J–L). To clarify its potential survival role, adiponectin protein levels were measured in conditioned medium of INS-1E cells, either untreated or treated with Ad-EVs or CK-EVs. Adiponectin was undetectable in β cells in all conditions (data not shown), whereas it was produced by 3T3-L1 adipocytes, which were used as positive control (2.193 ± 0.092 ng/mL, *n* = 3).

The effects of EVs in β cells were paralleled by changes in expression of CK genes. Indeed, while Ad-EVs had no effect on *Tnfa* (encoding TNF-α) and *Ifng* (encoding IFN-γ), CK-EVs upregulated the expression of both CKs, whereas *Il1b* (encoding IL-1β) was unchanged after treatment with both Ad-EVs and CK-EVs (Figure 3, M–O). These results indicate that in β cells Ad-EVs and CK-EVs differently modulate survival and ER stress pathways as well as expression of β cell genes, adipokines, and CKs.

### miRNAs are differentially expressed in Ad-EVs and CK-EVs.

**Figure 4:**
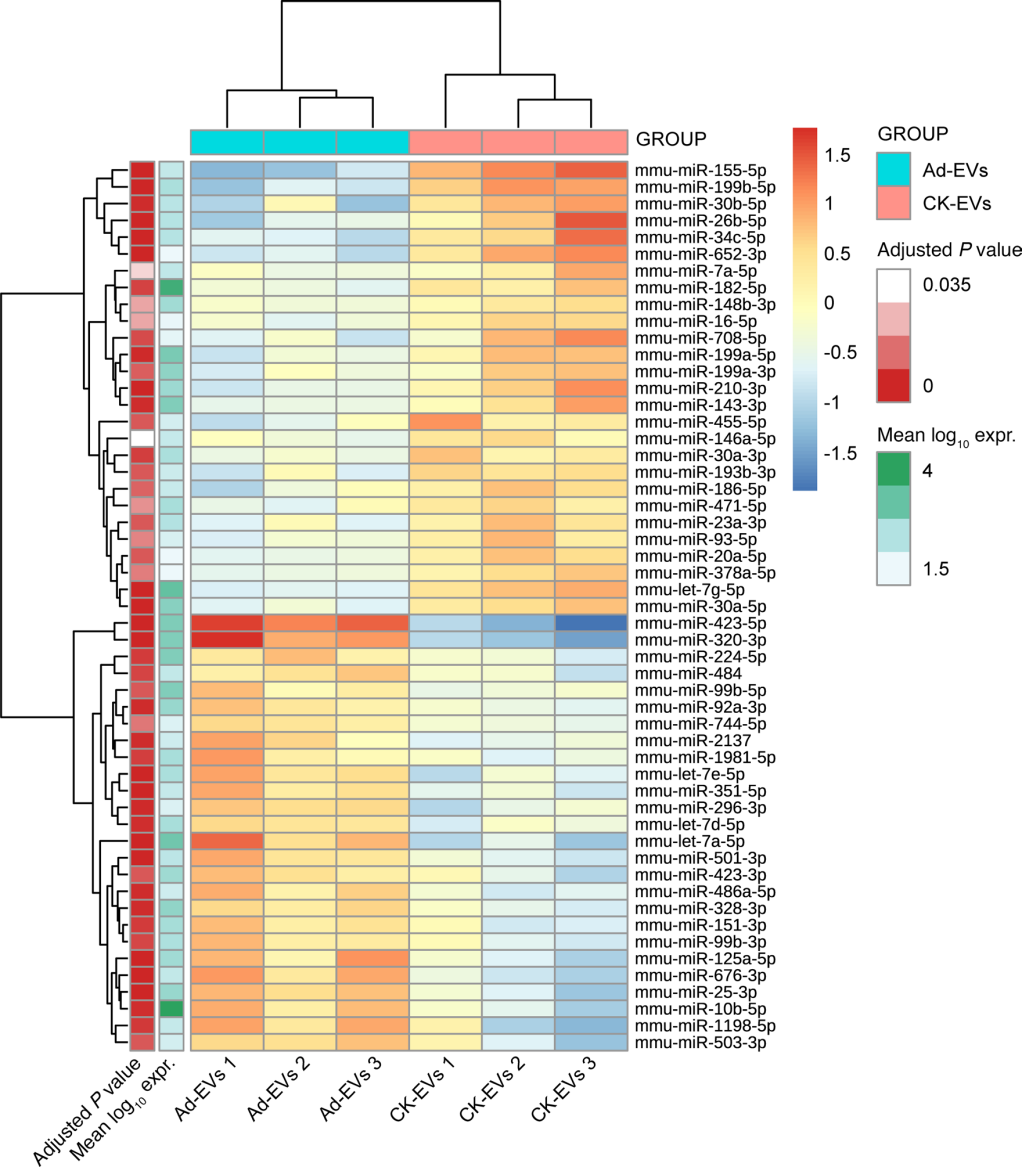
Heat map displaying the significantly differentially expressed miRNAs in Ad-EVs and CK-EVs. Each row represents a miRNA and each column a sample. The sample dendrogram, generated in an unsupervised way from the expression profiles, is shown at the top. The heat map color shows the log_2_ fold change normalized expression of each sample with respect to the mean expression (across samples) for each miRNA. The color scale on the right correspond to high (red), medium (yellow), and low (blue) expression, respectively.

To evaluate whether the EV cargo of small RNAs, in particular miRNAs, was different according to the treatment, the RNAs extracted from Ad-EVs and CK-EVs were sequenced for their small RNA content (small RNA-seq). EVs from 3 cultures for each treatment were sequenced. A median of 2.00 million raw reads were generated for the 6 samples analyzed. We found 53 miRNAs differentially expressed between Ad-EVs and CK-EVs. Of these, 27 were upregulated and 26 downregulated in CK-EVs compared with Ad-EVs (Figure 4 and Supplemental Table 5). The set of validated target genes for the miRNAs differentially expressed between Ad-EVs and CK-EVs was extracted with miRWalk database version 3 (34). 53 miRNAs had 422 validated target genes (213 unique genes), which were tested for overrepresentation using a gene set enrichment analysis (GSEA) program implemented in the new version of miRWalk. The Kyoto Encyclopedia of Genes and Genomes (KEGG) showed several pathways to be significantly enriched, including the AGE/RAGE signaling pathway in diabetic complications and Toll-like receptor and PI3K/Akt signaling pathways (Supplemental Table 6). The Gene Ontology (GO) database showed that several of the target genes were significantly enriched for biological processes implicated in systemic inflammation and insulin resistance (Supplemental Table 7). We further analyzed those validated miRNAs targeting genes that were altered in β cells after treatment with Ad-EVs and CK-EVs (Supplemental Table 8). Overall, we observed a substantial inverse expression between miRNAs and their target mRNAs. For example, whereas *TNFA* and *IFNG* mRNAs were increased in β cells treated with CK-EVs (Figure 3, M and N), the miRNAs targeting these genes (mmu-miR-296-3p, mmu-miR-298-5p, and mmu-miR-351-5p for TNF-α, and mmu-miR-125a-5p for IFN-γ) were downregulated (Supplemental Table 8). These results suggest that the downregulation of specific miRNAs allows an upregulation of their target genes and implicates a complex crosstalk between adipocytes and β cells, involving a miRNA-dependent posttranscriptional gene regulation in β cells.

### Ad-EVs, but not CK-EVs, increase cell survival in human pancreatic islets.

**Figure 5:**
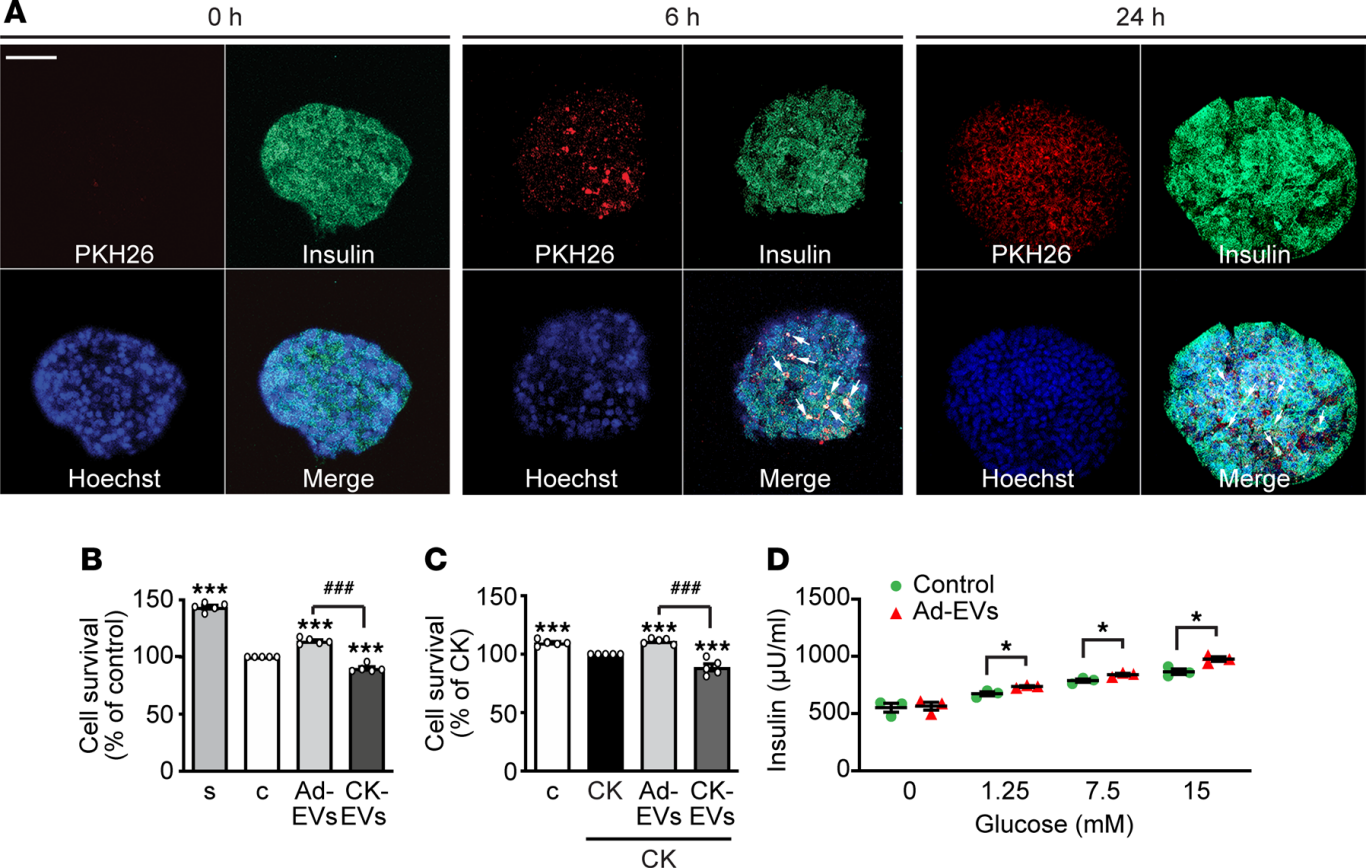
Effect of Ad-EVs and CK-EVs on survival and function of human pancreatic islets. (**A**) Representative confocal microscopy images of human pancreatic islets incubated with PKH26-labeled Ad-EVs at the times indicated. Ad-EVs are shown in red, DAPI-stained nuclei in blue, and insulin in green for β cells. Merge at 6 and 24 hours shows colocalization of Ad-EVs and insulin (yellow) in β cells, as indicated by arrows (scale bar: 50 μm). (**B**) Cell survival assessed by Alamar blue assay in islets cultured for 72 hours in normal medium with serum (s) or in serum-deprived medium alone (c) or with either Ad-EVs (1 × 10^8^/islet) or CK-EVs (1 × 10^8^/islet). (**C**) Cell survival in islets cultured in serum-deprived medium (control [c]) or exposed to cytokines (CK) (TNF-α/IFN-γ/IL-1β, 5 ng/mL each) and either untreated or treated with Ad-EVs or CK-EVs for 72 hours. Results for **B** and **C** are expressed as a percentage of control for **B** and percentage of CK for **C** (mean ± SEM) (*n* = 5). ***P < 0.001 vs. c (**B**) or CK (**C**); ^###^*P* < 0.001 by 1-way ANOVA and Tukey’s post hoc test. (**D**) Insulin secretion assessed by ELISA in pancreatic islets incubated with 2 mM glucose for 1 hour and then for a further 1 hour with the indicated concentrations of glucose, in the presence or absence of Ad-EVs (1 × 10^8^/islet) (mean ± SEM) (*n* = 4). *P < 0.05 at each glucose concentration, by Student’s 2-tailed *t* test.

The effects of Ad-EVs and CK-EVs were subsequently assessed in human pancreatic islets. Confocal microscopy analysis showed that incorporation of PKH26-labeled Ad-EVs increased at 6 and 24 hours, when the majority of islet cells were positive for the red dye (Figure 5A). Staining of Ad-EVs also revealed partial colocalization with insulin, indicating uptake by β cells. Furthermore, pretreatment with anti-CD29 antibody strongly reduced internalization of Ad-EVs (Supplemental Figure 2A). As for INS-1E β cells, we evaluated the role of Ad-EVs and CK-EVs on survival of islets cultured in the absence of serum or in the presence of CKs for 72 hours. Ad-EVs increased cell survival in both conditions, whereas CK-EVs exacerbated the detrimental effects of serum starvation and CKs (Figure 5, B and C). Similarly, Ad-EVs promoted, whereas CK-EVs reduced, cell survival after treatment with high glucose and palmitate (Supplemental Figure 2B). In addition, Ad-EVs increased GSIS at all the glucose concentrations tested (Figure 5D). Due to the limited availability of human islets, GSIS was not assessed in islets treated with CK-EVs.

### EVs from human lean AT exert positive effects, whereas EVs from obese AT show negative effects on survival and function of human β cells.

**Figure 6:**
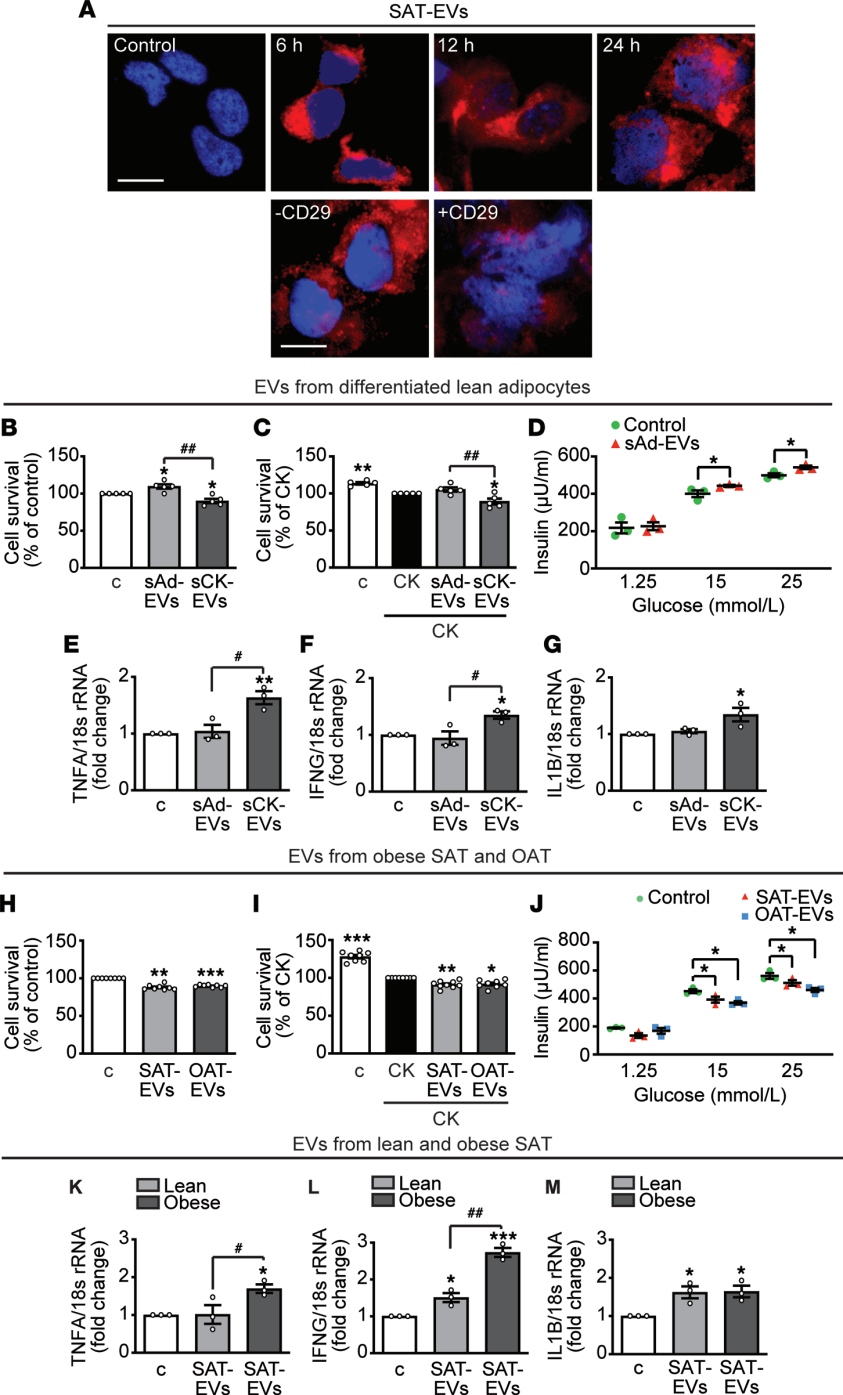
Effect of human lean and obese AT-derived EVs in human EndoC-βH3 β cells. (**A**) Representative fluorescence microscopy micrographs showing subcutaneous AT–derived EV (SAT-EV) internalization in EndoC-βH3 cells, incubated at the indicated times with SAT-EVs unlabeled (Control) or labeled with PKH26 (red dye) (scale bar: 10 μm). SAT-EV internalization (24 hours) in cells untreated or preincubated for 1 hour with CD29 antibody. Nuclei were stained blue with DAPI (scale bar: 10 μm). (**B**) Cell survival (MTT) in β cells untreated (control [c]) or treated for 24 hours with lean sAd-EVs (10 × 10^3^/cell) or EVs from lean subcutaneous differentiated adipocytes pretreated with TNF-α/IFN-γ/IL-1β (50, 25, and 2.5 ng/mL, respectively) (sCK-EVs). (**C**) Cell survival in β cells untreated or treated with sAd-EVs or sCK-EVs, then with TNF-α/IFN-γ/IL-1β (CK) (20, 20, and 1 ng/mL, respectively) 24 hours (mean ± SEM). *P < 0.05, **P < 0.01 vs. c (**B**) or vs. CK (**C**); ^##^*P* < 0.01 (*n* = 5), 1-way ANOVA and Tukey’s post hoc test. (**D**) Insulin secretion (ELISA) in β cells incubated for 1 hour with indicated concentrations of glucose with or without sAd-EVs (mean ± SEM). *P < 0.05, Student’s 2-tailed *t* test (*n* = 3). Real-time PCR for *TNFA* (**E**), *IFNG* (**F**), and *IL1B* (**G**) in cells untreated or treated with sAd-EVs or sCK-EVs (mean ± SEM). *P < 0.05, **P < 0.01 vs. c; ^#^*P* < 0.05, 1-way ANOVA and Tukey’s post hoc test (*n* = 3). Cell survival in β cells cultured in normal medium (**H**) or treated with CKs (as for **C**) (**I**), and with EVs from obese subcutaneous AT (SAT-EVs) or obese omental AT (OAT-EVs) (10 × 10^3^/cell) (mean ± SEM). * *P* < 0.05, **P < 0.01, **P < 0.001 vs. c (**H**) or vs. CK (**I**), 1-way ANOVA and Tukey’s post hoc test (*n* = 8). (**J**) Insulin secretion (ELISA) in EndoC-βH3 cells incubated as in **D** in the absence or presence of SAT-EVs or OAT-EVs (10 × 10^3^/cell) (mean ± SEM). *P < 0.05, Student’s 2-tailed *t* test (*n* = 3). Real-time PCR for *TNFA* (**K**), *IFNG* (**L**), and *IL1B* (**M**) mRNA in cells untreated or treated with lean or obese SAT-EVs 24 hours (mean ± SEM). *P < 0.05, ***P < 0.001 vs. c; ^##^*P* < 0.01, 1-way ANOVA and Tukey’s post hoc test (*n* = 3).

The effects of human AT-derived EVs were then assessed in the human pancreatic β cell line, EndoC-βH3. EVs from SAT-EVs were internalized by EndoC-βH3 cells, as demonstrated by PKH26 staining (Figure 6A, top), whereas pretreatment with anti-CD29 antibody strongly reduced their incorporation (Figure 6A, bottom). We then verified whether EVs from lean or obese individuals differently affected survival and function of β cells. EVs were isolated from both in vitro–differentiated adipocytes and ex vivo AT explants. In line with the previous results on 3T3-L1–derived EVs, EVs from in vitro–differentiated subcutaneous lean adipocytes (sAd-EVs) increased survival of β cells cultured in normal medium, compared with control cells (Figure 6B), whereas no effect was observed in β cells treated with CKs (Figure 6C). sAd-EVs also slightly promoted GSIS at 15 and 25 mM glucose (Figure 6D). Conversely, EVs from CK-treated lean subcutaneous differentiated adipocytes (sCK-EVs) reduced survival of β cells, cultured both without or with CKs (Figure 6, B and C). In addition, sCK-EVs, but not sAd-EVs, increased the expression of *TNFA*, *IFNG*, and *IL1B* (Figure 6, E–G). In line with their positive effects, sAd-EVs increased *PDX1* and *NKX6.1* mRNA in human β cells (Supplemental Figure 3, A and B) and upregulated *ADIPOQ* and *CFD* (adipsin) (Supplemental Figure 3, C and E). By contrast, sCK-EVs inhibited Pdx-1 gene expression and showed a mild effect on elevation of adiponectin, leptin, and adipsin (Supplemental Figure 3, C–E). Unlike EVs from sAd-EVs, EVs from lean SAT-EVs showed no effect on cell survival and GSIS (Supplemental Figure 3, F–H). We found 20 miRNAs differentially expressed between sCK-EVs and sAd-EVs, 17 upregulated, and 3 downregulated (Supplemental Figure 4 and Supplemental Table 9). GO analysis showed enrichment of target genes involved in biological processes, including negative regulation of cell cycle process, negative regulation of transcription and positive regulation of stress-activated protein kinase signaling cascade (Supplemental Table 10). However, no inverse expression between human miRNAs and their target mRNAs was observed (Supplemental Table 8).

We next investigated the impact of EVs obtained from AT explants of individuals with obesity. As opposed to EVs from differentiated lean adipocytes, EVs from both obese subcutaneous (SAT-EVs) and OAT (OAT-EVs) equally reduced survival of human β cells, untreated (Figure 6H) or treated with CKs (Figure 6I). Furthermore, they similarly blunted GSIS at 15 and 25 mM glucose (Figure 6J). We then analyzed the effect of obese and lean EVs on gene expression of CKs, β cell transcription factors, and adipokines in β cells, using only subcutaneous-derived EVs, because of the limited availability of OAT. *TNFA* mRNA was unchanged in β cells treated with lean SAT-EVs but increased by obese SAT-EVs, compared with untreated cells (Figure 6K). Similarly, although slightly increased by lean SAT-EVs, obese SAT-EVs strongly upregulated *IFNG* (Figure 6L), whereas *IL1B* levels were equally upregulated (Figure 6M). Lean, but not obese, SAT-EVs enhanced expression of *PDX1* and *NKX6.1* mRNAs (Supplemental Figure 3, I–J), consistent with the results previously observed for sAd-EVs (Supplemental Figure 3, A and B). Moreover, lean, more than obese, SAT-EVs increased adiponectin gene expression (Supplemental Figure 3K). Leptin was markedly upregulated by obese SAT-EVs, and upregulated far less by lean SAT-EVs, and adipsin was increased only by lean SAT-EVs (Supplemental Figure 3, L and M). Hence, similar to 3T3-L1–derived EVs, EVs from human subcutaneous differentiated adipocytes exert beneficial effects, whereas EVs from the same adipocytes pretreated with CKs are detrimental for β cells. Furthermore, EVs from obese, but not from lean, AT explants are harmful for β cells.

## Discussion

The adipokine-mediated crosstalk between adipocytes and other cell types, including immune cells and insulin-responsive cells, is a key mechanism regulating whole-body homeostasis (2). More recently, a relevant role in the communication between fat cells and other cell types has been attributed to AT-derived EVs (8); however, their effect on survival and function of pancreatic β cells remains unexplored. Here, we show that EVs from 3T3-L1 adipocytes increase survival and proliferation, reduce apoptosis, and positively influence GSIS in INS-1E β cells and human pancreatic islets, both those untreated or exposed to diabetogenic stimuli. In addition, by using anti-CD29 antibody, we demonstrate that uptake by β cells and internalization are essential for EVs to display their survival effects. Similarly, EVs from differentiated human lean adipocytes displayed beneficial activities in human EndoC-βH3 cells treated under the same stress conditions. By contrast, EVs from both 3T3-L1 and differentiated human lean adipocytes pretreated with CKs, as well as AT EVs from human obese individuals, reduced survival and function in rodent and human β cells. These effects were paralleled by differential expression of miRNAs in EVs, as well as modulation of survival and metabolic pathways in β cells and regulation of genes specific for β cells, adipokines, and CKs.

As previously demonstrated (11, 35, 36), we observed that EVs from both 3T3-L1 adipocytes and human SAT express markers for both EVs and adipocytes, such as adiponectin and leptin. Accordingly, fully functional AT was previously found to protect other metabolic organs from the harmful effect of dyslipidemia, in part through the beneficial role of adiponectin and adipsin (2). Moreover, EVs from lean mouse AT macrophages improved glucose tolerance and insulin sensitivity when administered to obese mice, whereas those from obese mice were harmful in lean mice (16). Our results also revealed that EVs from healthy and inflamed adipocytes differently regulate pathways essential for β cell survival, proliferation, and function, such as PI3K/Akt, its downstream effector GSK-3β, and ERK1/2 (27, 28). Ad-EVs and CKs also modulated the UPR, a pathway activated in response to the disruption of ER homeostasis, in an attempt to restore cellular equilibrium. The UPR includes different pathways, mediated by the action of 3 signaling proteins, PERK, IRE1α, and activating transcription factor 6 (ATF6). However, under irresolvable ER stress, the UPR switches from an adaptive to an apoptotic role (29–31). Importantly, ER stress and disrupted UPR have been implicated in the pathophysiology of both T1D and T2D (37). In addition, β cell exposure to proinflammatory CKs induces UPR responses, which in turn may potentiate inflammation and promote cell death (29, 31, 38). We found that CK-EVs promoted the phosphorylation of 2 main UPR proteins, PERK and its downstream effector eIF2α (PERK/eIF2α), and JNK, which can be phosphorylated by IRE1α, as well as CK receptors independently of the UPR (31, 38). Interestingly, JNK has been recently indicated as a key factor regulating the transition from adaptive to apoptotic UPR during ER stress induced by CKs (39). Conversely, Ad-EVs only weakly increased PERK/eIF2α activity, while having no effect on JNK. These findings can be explained by the fact that PERK/eIF2α can either reduce protein overload through decreasing general translational activity or initiate proapoptotic response via ATF4 and upregulation of the proapoptotic UPR effector CHOP. Thus, we hypothesize that Ad-EVs may induce a compensatory pathway, while CK-EVs diverts PERK/eIF2α signaling toward the ATF4-mediated increase in CHOP expression. This assumption is further sustained by our results on Chop mRNA, which was increased by CK-EVs but not Ad-EVs. In addition, only CK-EVs promoted the phosphorylation of JNK, the IRE1α-mediated activity of which is required for CHOP promoter activation and expression (38, 39).

A crucial role for GSK-3β on insulin secretion has been recently reported, involving a Pdx1-dependent mechanism, as well as glucokinase, Glut2, and Nkx.6.1 (27, 40). Accordingly, we found that Ad-EVs, but not CK-EVs, increased the phosphorylation, i.e., reduced the activity, of GSK-3β; in addition, EVs from healthy adipocytes and lean SAT, but not from CK-treated adipocytes or obese SAT explants, upregulated Pdx1 and Nkx6.1. These transcription factors are essential for β cell differentiation and maintenance of adult β cell function, and their activity has been found perturbed in conditions of metabolic stress (32). Furthermore, many studies have demonstrated an association between high adiponectin levels and increased insulin sensitivity, β cell function, and survival, whereas obesity and elevation of inflammatory CKs in AT have been linked to reduced adiponectin production (2, 3, 41). In keeping with these findings, both EVs from healthy 3T3-L1 and lean SAT-derived differentiated adipocytes strongly upregulated the expression of adiponectin in INS-1E and EndoC-βH3 cells, whereas adiponectin expression was low after exposure to CK-EVs, suggesting horizontal gene transfer to β cells. However, we could not find detectable levels of adiponectin protein in conditioned medium of INS-1E β cells challenged with Ad-EVs, indicating that the mRNA levels transferred by EVs are not translated into a sufficient amount of protein to produce an effect in these cells. Similar changes were observed for adipsin, an adipokine of the complement system that stimulates GSIS in β cells and with levels that have been found to be reduced in patients with T2D with β cell failure (33). Interestingly, EVs from lean SAT explants increased adiponectin and adipsin as well as Pdx-1 and Nkx6.1 mRNA in human β cells, despite having no effect on β cell survival. Beside the fact that the protein levels of adiponectin are likely not detectable in human β cells, as observed in INS-1E β cells treated with Ad-EVs, SAT-EVs derive from a mixed cell population, including mesenchymal stem cells, fibroblasts, and immune cells, in addition to mature adipocytes (42), which would generate a cargo different from that of 3T3-L1 adipocytes or human differentiated adipocytes. Lean SAT-EVs also slightly upregulated IFN-γ and IL-1β but not TNF-α in human β cells, whereas obese SAT-EVs increased the mRNA levels of all CKs and leptin but had no effect on β cell genes. Indeed, it is known that CKs, particularly TNF-α, but also leptin, which inhibits GSIS and with production that is increased in obese SAT, released by obese AT are harmful for β cells (2, 4, 6).

In line with our findings, injection of AT-derived EVs from *ob/ob* mice into mice fed with both normal and high-fat diet induced macrophage activation and upregulation of inflammatory CKs, suggesting that EV cargo is altered in obesity and promotes inflammatory responses either locally or at distant sites (15). Furthermore, EVs from insulin-resistant muscles of palmitate-treated mice influence gene expression and proliferation, but not GSIS, in mouse β cells, contributing to β cell mass adaptation during insulin resistance. However, although internalized in pancreatic islets, these EVs were found to be incorporated by other cells types before reaching the pancreas (25). At variance with our findings, Kranendonk et al. reported that EVs from both human in vitro–differentiated SAT adipocytes and SAT and OAT explants induce the differentiation of monocytes to inflammatory macrophages (11); yet, these EVs derived from both normal and insulin-resistant individuals and, thus, had an overall altered cargo. Dysregulated insulin signaling was also observed in the liver and muscle cells after treatment with EVs from SAT and OAT explants of obese patients, overweight patients, and patients with T2D (10).

The results of this study also suggest a role for miRNAs, as we observed their differential expression in EVs from CK-treated adipocytes, compared with untreated adipocytes. miRNAs are noncoding RNAs, acting on target mRNAs to induce their degradation or inhibition of translation. Interestingly, miRNAs are among the main components of EV cargo and can modulate the function of neighboring or distant recipient cells (7). Moreover, AT-derived EVs are an important source of miRNAs, and their levels may change in diseases with altered fat mass and function, such as obesity and diabetes (13, 43). miRNAs are also potent regulators of β cell biology (44) through the promotion of β cell dysfunction and death under metabolic stress conditions, and their dysregulation has been linked to the development of T1D and T2D (20, 23, 45). We observed that preincubation of 3T3-L1 adipocytes and human subcutaneous differentiated lean adipocytes with inflammatory CKs alters miRNA profile in EVs. These changes were paralleled by an enrichment in metabolic pathways in EVs derived from CK-treated adipocytes, compared with untreated adipocytes, and an increase in target genes involved in systemic inflammation and insulin resistance. miR-155-5p was the most upregulated miRNA, in agreement with previous reports showing increased miR-155-5p levels in TNF-α– or palmitic acid–treated 3T3-L1 adipocytes (14, 46) and its important role on glucose and lipid metabolism and β cell function (16, 23, 47, 48). In addition, miR-30a-5p, increased here in CK-EVs, has been linked to GSIS and β cell dysfunction, and miR-146 with CK-induced apoptosis in β cells (44). Among the downregulated miRNAs in CK-EVs, miR-320-3p and miR-501 have been related to the positive effects of antidiabetic drugs in β cells (49). In both 3T3-L1 and human adipocyte EVs, we found differential expression of miR-7, which is implicated in inhibition of GSIS and which was found at increased levels in cadaveric islets of diabetic donors (44). Thus, we cannot exclude a role for miRNAs in the effects of adipocyte-derived EVs in β cells; however, miRNAs are likely not solely responsible. It is possible that they are part of a complex mechanism involving other components of EV cargo.

In conclusion, herein we show that adipocyte-derived EVs positively or negatively influence survival, proliferation, and function of pancreatic β cells and human pancreatic islets in vitro, depending on the state of adipocytes and the origin of AT. Translated in vivo, healthy AT would evoke beneficial effects, whereas in pathological conditions such as obesity and insulin resistance, the crosstalk between adipocytes and pancreatic β cells would generate a negative loop, further amplifying the progression of insulin resistance and β cell dysfunction. Understanding the molecular mechanisms involved in EV actions would help the design of new strategies to prevent β cell loss and diabetes. Interestingly, the survival effects observed in human pancreatic islets suggest that EVs may act on other islet cell types in addition to β cells; however, this intriguing hypothesis requires additional investigation. In 1996, Kieffer et al. proposed the concept of adipoinsular axis, which was initially identified with the effects of leptin in β cells and then extended to the biological activities of many adipokines (2, 50). In agreement with that view, our in vitro findings suggest the existence of a functional adipoinsular axis involving EVs and their cargo as possible regulators of β cell survival and function.

## Methods

### Cell culture.

3T3-L1 murine preadipocytes (ATCC) were maintained in DMEM (MilliporeSigma) supplemented with 10% FCS (Life Technologies). The cells were cultured and differentiated into adipocytes as described previously (51). Briefly, 2 days after confluence (day 0), cells were treated with differentiation medium (5 μg/mL insulin, 1 μM dexamethasone, and 0.5 mM 3-Isobutyl-1-methylxanthine [IBMX]) in DMEM supplemented with 10% FBS (MilliporeSigma). At day 2, cells were switched to DMEM with 10% FBS and 1 μg/mL insulin (MilliporeSigma) until day 8. INS-1E rat insulinoma β cells (AddexBio), used for no more than 10 passages, were cultured in RPMI-1640 (MilliporeSigma) with 0.02 mM 2-mercaptoethanol and 10% FBS (27). The conditionally immortalized human pancreatic β cell line EndoC-βH3 was purchased from Univercell-Biosolutions and cultured according to the manufacturer’s instructions in OPTl β1 media containing 10 μg/mL puromycin (52). The cells were seeded onto βCOAT-treated tissue culture flasks at 7 × 10^4^ cells/cm^2^. Inducible excision of CRE-mediated immortalizing transgenes for GSIS experiments was performed with addition of 1 μM tamoxifen (MilliporeSigma) for 21 days. 3T3-L1, INS-1E, and EndoC-βH3 cells were routinely checked by RT-PCR to exclude mycoplasma contamination. All the cells were cultured at 37°C in a 5% CO_2_ humidified atmosphere.

### Human pancreatic islets.

Human pancreatic islets were isolated from heart-beating cadaveric organ donors in the Pancreatic Islet Processing Unit of the Diabetes Research Institute at the San Raffaele Scientific Institute (Supplemental Table 1) (27). Islet purity was assessed as the percentages of endocrine clusters positive for dithizone staining (range, 80%–90%). After isolation, islets (10,000) were cultured in CMRL medium (MilliporeSigma), as previously described (27).

### Human individuals.

SAT and OAT explants from 18 obese nondiabetic individuals (13 women and 5 men, with an average BMI of 39.4 ± 3.7) were obtained during laparoscopic surgery for nonmalignant diseases at the Department of Surgical Sciences, University of Turin. SAT explants were also obtained from 9 lean adult individuals (3 women and 6 men, with an average BMI of 25.7 ± 2) during elective plastic surgery of at the Department of Reconstructive and Aesthetic Plastic Surgery, University of Turin.

### Isolation and differentiation of human adipocytes.

Adipocyte isolation from SAT explants of lean subjects was performed as described previously (51). Preadipocytes were cultured in DMEM/F12 supplemented with 10% FBS until confluence. Then, preadipocyte culture medium was changed to DMEM with 10% FBS, 15 mM HEPES, 33 μM biotin, 17 μM pantothenate, 10 μg/mL transferrin, 5 μg/mL human insulin, 1 μM dexamethasone, and 0.5 mM IBMX for 3 days to initiate differentiation. At day 4, dexamethasone and IBMX were removed from the medium and the cells were cultured for a further 21 days before experiments.

### Isolation and characterization of EVs.

3T3-L1 and human adipocytes were cultured in RPMI without serum for 24 hours in the presence or absence of CKs (TNF-α/IFN-γ/IL-1β; 50, 25, and 2.5 ng/mL, respectively; PeproTech EC Ltd.). SAT-EVs and OAT-EVs were isolated from fresh human AT specimens. Samples were rinsed twice with PBS 1× to remove blood cells, mechanically minced into small pieces, and maintained in RPMI medium without serum or growth factors for 24 hours at 37°C in a 5% CO_2_ humidified atmosphere. EVs were collected from supernatants. After being centrifuged at 3000*g* for 20 minutes and microfiltered over a 0.22 μm filter (MilliporeSigma) to remove debris, cell-free supernatants derived from cell cultures and human AT were ultracentrifuged at 100,000*g* using a SW70Yi rotor (Beckman Coulter Optima L-90K ultracentrifuge) for 2 hours at 4°C. EVs were either used fresh or stored at –80°C after resuspension in RPMI supplied with 1% DMSO (v/v). Frozen EVs were washed and pelleted under 100,000*g* ultracentrifugation to remove DMSO before experiments on cells, as previously described (17). EV number and size distribution analysis was performed using a NanoSight LM10. The particles in the samples were illuminated using a laser light source at 405 nm, and the scattered light was captured by camera and analyzed using NTA. NTA automatically tracked and sized more than 200 particles, according to Brownian motion and the diffusion coefficient. Results are displayed as number per mL and as frequency size distribution graphs, outputted to a spreadsheet. Characterization of EVs was performed according to the criteria suggested by the ISEV (53). Briefly, purified EVs were observed by TEM and analyzed by Western blot for the expression of Alix, CD63, and CD9. Adipocyte markers, such as adiponectin, leptin, FABP4, PPARγ-1, and PPARγ-2, were assessed by RT-PCR and Western blot.

### TEM.

TEM was performed on 3T3-L1–derived EVs (Ad-EVs) and SAT-EVs isolated by ultracentrifugation, resuspended in PBS 1×, placed on 200 mesh nickel formvar carbon-coated grids (Electron Microscopy Science), and left to adhere for 20 minutes. Grids were then incubated with 2.5% glutaraldehyde, containing 2% sucrose, and EVs were negatively stained with NanoVan (Nanoprobes), after being washed in distilled water, and observed under a Jeol JEM 1010 electron microscope.

### EV internalization in INS-1E and EndoC-βH3 β cells.

The internalization of EVs into INS-1E and EndoC-βH3 β cells was evaluated using fluorescent microscopy. A pool of EVs was labeled with red fluorescent PKH26 dye (2 μL/mL; MilliporeSigma) for 30 minutes at 37°C and EVs were then washed and ultracentrifuged at 100,000*g* at 4°C for 1 hour. EV pellets were resuspended in RPMI and used at 5 × 10^3^ EV/target cell concentration to INS-1E or EndoC-βH3 β cells and preincubated or not with the anti-CD29 blocking monoclonal antibody (1 μg/mL; Santa Cruz Biotechnology) to assess their internalization. Cells were fixed with 10% paraformaldehyde, and nuclei were stained with DAPI (1:5000; MilliporeSigma) for 10 minutes at 4°C. Images were taken using a Leica DM200 fluorescent microscope and a Leica DFC340 FX camera, and analysis performed with a Leica Suite image analysis software.

### EV internalization in human pancreatic islets.

The internalization of EVs into human pancreatic islets was evaluated using confocal microscopy (LSM5-PASCAL; Zeiss). 1 × 10^8^ EVs were labeled with red fluorescent PKH26 dye (2 μL/mL) for 30 minutes at 37°C and then washed and ultracentrifuged at 100,000*g* at 4°C for 1 hour. The EV pellet was resuspended in CMRL medium and added to human pancreatic islets (1 × 10^8^ EVs/islet) up to 24 hours to detect internalization. After fixation in 4% paraformaldehyde, human pancreatic islets were stained overnight at 4°C with rabbit polyclonal anti-insulin antibody (1:200; Abcam). The day after, the cells were incubated for 1 hour at room temperature with Alexa Fluor 488–conjugated goat anti-rabbit antibody (1:450; Invitrogen). Nuclei were stained with Hoechst 33258 (1:1000; MilliporeSigma) for 10 minutes at 4°C. Absence of primary antibody was used as negative control. *Z* stack confocal microscopy EC images were also obtained.

### Cell survival and proliferation.

INS-1E β cells were seeded in 96-well plates at 3 × 10^3^ cells/well and cultured for 48 hours, serum starved for 24 hours, and incubated with the different stimuli for a further 24 hours. Cell survival and proliferation were assessed using the MTT assay (MilliporeSigma) and 5-bromo-2-deoxyuridine (BrdU) incorporation ELISA kit (Roche Diagnostics), respectively, as previously described (27, 51). Human pancreatic islets were seeded in 96-well plates (3 islets/well) and cultured for 48 hours, serum starved for 24 hours, and incubated with the different stimuli for a further 72 hours. Cell survival was assessed by Alamar blue assay (MilliporeSigma), as previously described (27). EndoC-βH3 β cells were seeded in 96-well plates at 10 × 10^3^ cells/well, cultured for 48 hours, and incubated with different stimuli for a further 24 hours in OPTlβ1 media. Cell survival was assessed by MTT assay. Absorbance was assessed by spectrophotometry at 570 nm for MTT and Alamar and at 450 nm for BrdU, using a LT-4000 microplate reader (Euroclone).

### Caspase-3 activity.

INS-1E β cells were seeded in 6-well plates at 5 × 10^4^ cells/well. After 48 hours the cells were serum starved for 24 hours and incubated with Ad-EVs or EVs obtained from CK-EVs for an additional 24 hours. Caspase-3 activity was assessed by the Caspase-3 Colorimetric Assay Kit (BioVision) in cell lysates, according to the manufacturer’s instructions, and analyzed by colorimetric detection at 450 nm absorbance with a LT-4000 microplate reader (Euroclone).

### Western blotting.

3T3-L1 adipocytes, INS-1E β cells, SAT, Ad-EVs, and SAT-EVs were lysed in RIPA buffer (MilliporeSigma), and protein concentrations were calculated as previously described (27). Proteins (70 μg) were resolved in 11% SDS-PAGE (15% for CD9, leptin, and FABP4) and transferred to a nitrocellulose membrane. After blocking with 5% BSA in Tris-buffered saline with 0.1% Tween (MilliporeSigma) for 1 hour at room temperature, membranes were incubated overnight at 4°C with the specific antibody (Alix, CD63, CD9, adiponectin, leptin, FABP4, PPARγ, phospho-Akt (P-Akt) (Ser473), P-glycogen synthase kinase 3β (P-GSK-3β) (Ser9), P-ERK1/2 (Thr202/Tyr204), P-PERK (Thr980), P-eIF2α (Ser51), P-SAPK/JNK (Thr183/Tyr185), and actin (dilution 1:1000; leptin, FABP4, PPARγ, and actin, 1:500). Blots were reprobed with the respective total antibodies or actin for normalization. Immunoreactive proteins were visualized using horseradish peroxidase–conjugated goat anti-mouse or goat anti-rabbit (1:4000) antibodies (SouthernBiotech) by enhanced chemiluminescence using ChemiDoc XRS (Bio-Rad). Each experiment was performed in triplicate. Densitometric analysis was carried out with Quantity One software (Bio-Rad). (See Supplemental Table 2 for detailed information on antibodies.)

### GSIS.

GSIS in INS-1E β cells and human pancreatic islets was performed as previously described (27). Briefly, INS-1E β cells (5 × 10^5^ cells) and human pancreatic islets (*n* = 3) were serum starved for 24 hours and then incubated for 1 hour at 37°C in HEPES-buffered Krebs-Ringer bicarbonate buffer (MilliporeSigma) containing 0.5% BSA and 2 mM glucose (MilliporeSigma). The medium was changed, and the cells were incubated for 1 hour in Krebs-Ringer bicarbonate buffer/0.5% BSA containing 1.25, 7.5, and 15 mM glucose, with or without Ad-EVs or CK-EVs (10 × 10^3^/cell). Insulin release from INS-1E β cells and human pancreatic islets was quantified by the rat Insulin ELISA kit (Tebu-Bio) and human IRMA Insulin kit (Pantec), respectively, following the manufacturer’s instructions. After excision of CRE transgenes by tamoxifen (1 μM; MilliporeSigma) for 21 days, GSIS in EndoC-βH3 β cells was evaluated according the manufacturer’s instructions. Briefly, EndoC-βH3 β cells were incubated with βKREBS BSA buffer for 1 hour, then for an additional hour with 1.25, 15, and 25 mM glucose in either absence or presence of sAd-EVs and SAT-EVs or OAT-EVs (10 × 10^3^/cell). Insulin secretion was assessed by the Insulin ELISA kit (Mercodia) following the manufacturer’s instructions.

### RT-PCR and real-time PCR.

Total RNA isolation and reverse transcription to cDNA (3 μg RNA for RT-PCR and 1 μg RNA for real-time PCR) from 3T3-L1 adipocytes, INS-1E cells, EndoC-βH3 cells, SAT, Ad-EVs, and SAT-EVs treated with TRIzol reagent (Life Technologies) were performed as described previously (27). For RT-PCR, 9 μL cDNA was amplified in a 50 μL volume using AmpliTaq Gold Polymerase in a GeneAmp PCR System (PerkinElmer). Amplifications were assessed in the following conditions: 95°C for 30 seconds, annealing for 30 seconds (55°C for adiponectin and FABP4; 57°C for leptin; 56°C for PPAR-γ1; and 54°C for PPAR-γ2), 72°C for 60 seconds, and 72°C for 7 minutes for the elongation step. The final PCR products for adiponectin (mouse,122 bp; human,110 bp), leptin (mouse,181 bp; human, 211 bp), FABP4 (mouse,151 bp; human,154 bp), PPAR-γ1 (mouse, 214 bp; human, 150 bp), PPAR-γ2 (mouse, 350 bp; human, 123 bp), and 18S rRNA (120 bp) were separated by 2% agarose gel electrophoresis and visualized by ethidium bromide staining. 3T3-L1 adipocytes and SAT were used as positive control. 18S rRNA served as internal control; the negative control consisted of no RNA. For real-time PCR, cDNAs were treated with DNA-free DNase (Life Technologies), and the reaction was performed with 50 ng cDNA, 100 nM of each primer, and IQ-SYBR-green Mastermix (Bio-Rad) using the ABI-Prism 7300 (Applied Biosystems). 18S rRNA was used as endogenous control. Relative quantification was performed using the comparative Ct (^2−^ΔΔCt) method. Primers were designed with Primer 3 Software (http://www.primer3.org/), and sequences and details are reported in Supplemental Tables 3 and 4.

### Adiponectin secretion.

Adiponectin levels were measured in INS-1E β cell–concentrated (18-fold) conditioned medium using the Adipoq ELISA Kit (Abnova, Tebu-bio s.r.l), following the manufacturer’s instructions. 3T3-L1 adipocyte–conditioned medium was used as positive control.

### Library preparation for small RNA-Seq.

Total RNA from 3T3-L1–derived Ad-EVs and CK-EVs, and sAd-EVs and sCK-EVs from subcutaneous human differentiated adipocytes, was extracted with mirVana kit (Ambion, Life Technologies) according to the manufacturer’s instructions. RNA was quantified by Qubit 2.0 Fluorometer with the Qubit microRNA Assay Kit (Invitrogen) according to MIQE guidelines (http://miqe.gene-quantification.info/). Small RNA transcripts were converted into barcoded cDNA libraries with the NEBNext Multiplex Small RNA Library Prep Set for Illumina (New England BioLabs Inc.), as previously described (54) and according to the manufacturer’s protocol (protocol E7330, New England BioLabs Inc.). Libraries were pooled together (24 plex) and subjected to the Illumina sequencing pipeline, passing through clonal cluster generation on a single-read flow cell (Illumina Inc.) and 75 cycles of sequencing-by-synthesis on the Illumina Next-Seq 500. Computational miRNA data analysis was performed following the previously described optimized workflow (54). Raw sequencing data were deposited on Gene Expression Omnibus (GSE158654). Further details are provided in the Supplemental Methods.

### Statistics.

Results are expressed as mean ± SEM. Statistical significance was determined using 1-way ANOVA with Tukey’s or Dunnett’s post hoc analysis for multiple groups or unpaired 2-tailed Student’s *t* test to compare 2 groups when appropriate. Wald’s test with Benjamini-Hochberg false discovery rate correction for multiple testing was used for RNA-Seq. Statistical analysis was performed with GraphPad Prism 5.0 Software and R package DESeq2 1.22.2 (http://www.bioconductor.org/packages/release/bioc/html/DESeq2.html). Expression levels of significantly differently expressed miRNAs were displayed using the R package pheatmap. miRNA functional enrichment analysis was performed using EnrichR web tool (https://maayanlab.cloud/Enrichr/, ref. 55) on the list of validated miRNA targets annotated in the miRWalk 2.0 database (http://mirwalk.umm.uni-heidelberg.de/, ref. 34). Significance was established at *P* < 0.05.

### Study approval.

Human pancreatic islets were isolated from heart-beating cadaveric organ donors in the Pancreatic Islet Processing Unit of the Diabetes Research Institute at the San Raffaele Scientific Institute. The use of human pancreatic islets (islet preparations discarded from clinical use) was approved by **the** institutional review board of the San Raffaele Scientific Institute under the “European Consortium for Islet Transplantation (ECIT), human islet distribution program,” supported by the JDRF (3-RSC-2016-160-I-X). The study protocol for the use of human AT explants was approved by the ethics committee of Azienda Ospedaliero-Universitaria Città della Salute e della Scienza di Torino, Turin, Italy (CS/100 – protocol 12175; February 4, 2014)], and all the individuals provided informed written consent before surgery.

## Author contributions

RG conceived the study. RG, IG, GM, and GC provided methodology. IG, BP, E. Gargantini, GG, GB, AF, DB, NC, EF, MCD, GT, GZ, and SB provided investigation. MAB, MM, SA, RN, and LP provided resources. GB and BP provided formal analysis. RG wrote the original draft. RG, IG, and BP reviewed and edited the manuscript. RG, E. Ghigo, and GM acquired funding. RG, MFB, RML, JPC, HO, GM, JMFP, E. Ghigo, and GC supervised the study.

## Supplementary Material

Supplemental data

## Version 1 02/04/2021

In-Press Preview

## Version 2 03/08/2021

Electronic publication
