# Supplementary material for: Adipocyte-derived extracellular vesicles regulate survival and function of pancreatic β cells
Source: JCI Insight. 2021 Mar 8;6(5):e141962. doi: 10.1172/jci.insight.141962 (PMC8021102; doi:10.1172/jci.insight.141962)
Supplement: Supplemental data [file jciinsight-6-141962-s064.pdf]

Gesmundo I et al.

Adipocyte-derived extracellular vesicles regulate survival and function of pancreatic β-cells

Supplemental Materials and Methods

Library Preparation For small RNA-Seq

Total RNA from 3T3-L1-derived Ad-EVs and CK-EVs; human sAd-EVs and sCK-EVs, and human lean SAT-EVs and obese SAT-EVs was extracted with mirVana kit (Ambion, LifeTechnologies, Milan, Italy) according to manufacturer's instructions. RNA was quantified by Qubit® 2.0 Fluorometer with Qubit® microRNA Assay Kit (Invitrogen, Life Technologies, Carlsbad, CA, USA) according to MIQE guidelines (http://miqe.gene-quantification.info/). Small RNA transcripts were converted into barcoded cDNA libraries with the NEBNext Multiplex Small RNA Library Prep Set for Illumina (New England BioLabs Inc., Ipswich, USA) as previously described [1]. Multiplex adaptor ligations, reverse transcription primer hybridization, reverse transcription reaction and PCR amplification were performed according to the protocol for library preparation (Protocol E7330, New England BioLabs Inc., Ipswich, USA). Next, the obtained cDNA constructs were purified with the QIAQuick PCR Purification Kit (Qiagen, Hilden, Germany) following the modifications suggested by the NEBNext Multiplex Small RNA Library Prep Protocol and loaded on the Bioanalyzer 2100 (Agilent, Santa Clara, CA, USA) using the DNA High Sensitivity Kit (Agilent, Santa Clara, CA, USA) according to the manufacturer's protocol. Libraries were pooled together (24plex) and further purified with a gel size selection. A concluding Bioanalyzer 2100 run with the High Sensitivity DNA Kit (Agilent, Santa Clara, CA, USA) that allows the analysis of DNA libraries regarding size, purity and concentration, completed the workflow of library preparation. The obtained sequence libraries were subjected to the Illumina sequencing pipeline, passing through clonal cluster generation on a single-read flow cell (Illumina Inc., San Diego, CA, USA) and 75 cycles sequencing-by-synthesis on the Illumina Next-Seq 500 (Illumina Inc., San Diego, CA, USA). Computational analyses for miRNA data were performed following the previously described optimized workflow [2] . Reads shorter than 14 nucleotides were discarded from the analysis; adapter sequences were trimmed from the remaining reads by the Cutadapt software (http://journal.embnet.org/index.php/embnetjournal/article/ view/200). The trimmed reads were mapped against the mouse or human precursor miRNA sequences downloaded from miRBase (Release 21) by the Shrimp aligner. Mapped reads were assigned to the correct arm

by their alignment position in the precursor and a matrix of integer values called count matrix was created. A maximum of three mismatches between reads and reference miRNA sequences was allowed for the analysis. A median of 2.00 and 4.90 million raw reads were generated for the six mouse and six human samples, respectively. After adapter trimming of raw reads and removal of reads with less than 14 nucleotides a median of 1.45 and 3.66 million clean reads for mouse and human samples were obtained, respectively. Clean reads were mapped to a reference of murine or human miRNA sequences collected from miRBase (release 21), with a median of 0.24 and 1.14 million correctly mapped reads, respectively. Expression matrices were computed by counting mapped reads for 119 and 245 miRNAs, respectively. miRNA functional enrichment analysis was performed using EnrichR web tool [3] on the list of validated miRNA targets annotated in miRWalk 2.0 database [4]. Raw sequencing data were deposited on Gene Expression Omnibus with the identifier GSE158654 (token for Reviewer access: uxkjgeqqppojlsx).

References

1. Ferrero, G., Cordero, F., Tarallo, S., Arigoni, M., Riccardo, F., Gallo, G., et al.
Small non-coding RNA profiling in human biofluids and surrogate tissues from healthy individuals: description of the diverse and most represented species
Oncotarget, 9 (3) (2018), pp. 3097-3111.
2. Sabo, A.A., Birolo, G., Naccarati, A., Dragomir, M.P., Aneli, S., Allione, A., et al.
Small Non-Coding RNA Profiling in Plasma Extracellular Vesicles of Bladder Cancer Patients by Next-Generation Sequencing: Expression Levels of miR-126-3p and piR-5936 Increase with Higher Histologic Grades
Cancers (Basel), 12 (6) (2020), pp.
3. Kuleshov, M.V., Jones, M.R., Rouillard, A.D., Fernandez, N.F., Duan, Q., Wang, Z., et al.
Enrichr: a comprehensive gene set enrichment analysis web server 2016 update
Nucleic Acids Res, 44 (W1) (2016), pp. W90-97.
4. Dweep, H., Gretz, N.
miRWalk2.0: a comprehensive atlas of microRNA-target interactions
Nat Methods, 12 (8) (2015), pp. 697.

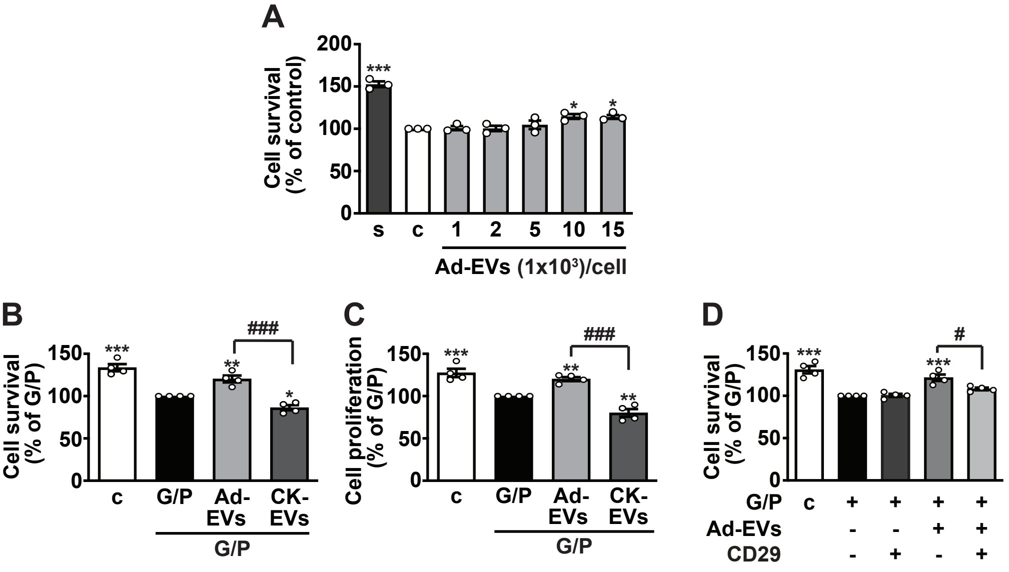

Figure S1. Effects of Ad-EVs and CK-EVs in INS-1E β-cells exposed to glucolipotoxicity. (A) Cell survival assessed by MTT in cells cultured in medium with serum (s) or in serum-deprived medium for 12 h (c, control), then treated for 24 h with the indicated doses of Ad-EVs. Results, expressed as percent of c, are means ± SEM. **P* < 0.05, ****P* < 0.001 vs. c by 1-way ANOVA and Dunnett's post-hoc test (n=3). **(B-C)** Cell survival and proliferation (MTT and BrdU) in cells serum-deprived (c, control) for 12 h, then treated for 24 h with high glucose (30 mM) and palmitate (0.4 mM) (G/P), and with either Ad-EVs or CK-EVs, that were added 40 min prior to G/P. Results, expressed as percent of G/P, are means ± SEM. **P* < 0.05, ***P* < 0.01, ****P* < 0.001 vs. G/P; $^{###}$*P* < 0.001 by 1-way ANOVA and Tukey's post-hoc test (n=4). **(D)** Cell survival in cells cultured for 24 h with or without G/P, Ad-EVs and CD29 blocking antibody. Results are means ± SEM. ****P* < 0.001 vs. G/P alone; $^{#}$*P* < 0.05 by 1-way ANOVA and Tukey's post-hoc test (n=4).

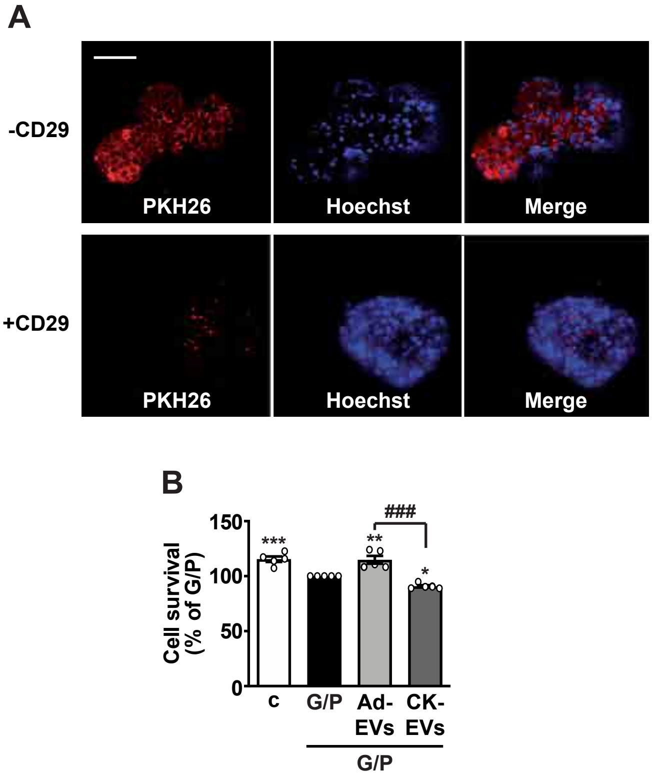

Figure S2. Effects of Ad-EVs and CK-EVs on survival of human pancreatic islets exposed to glucolipotoxicity. (A) Representative confocal microscopy images showing internalization of PKH26-labeled Ad-EVs (red) by human pancreatic islets at 24 h, in either absence (-CD29) or presence (+CD29) of anti-CD29 antibody. Nuclei were stained in blue with Hoechst 33258 (scale bar: 10 µm). **(B)** Cell survival assessed by Alamar blue assay in islets cultured for 72 h in serum-deprived medium, alone (c) or in the presence of high glucose (30 mM) and palmitate (0.4 mM) (G/P), and with either Ad-EVs or CK-EVs (1×10^8 EVs/islet), that were added 40 min prior to G/P. Results are means ± SEM (n=5). $^*P < 0.05$, $^{**}P < 0.01$,$^{***}P < 0.001$ vs. G/P; $^{###}P < 0.001$ by 1-way ANOVA and Tukey's post-hoc test.

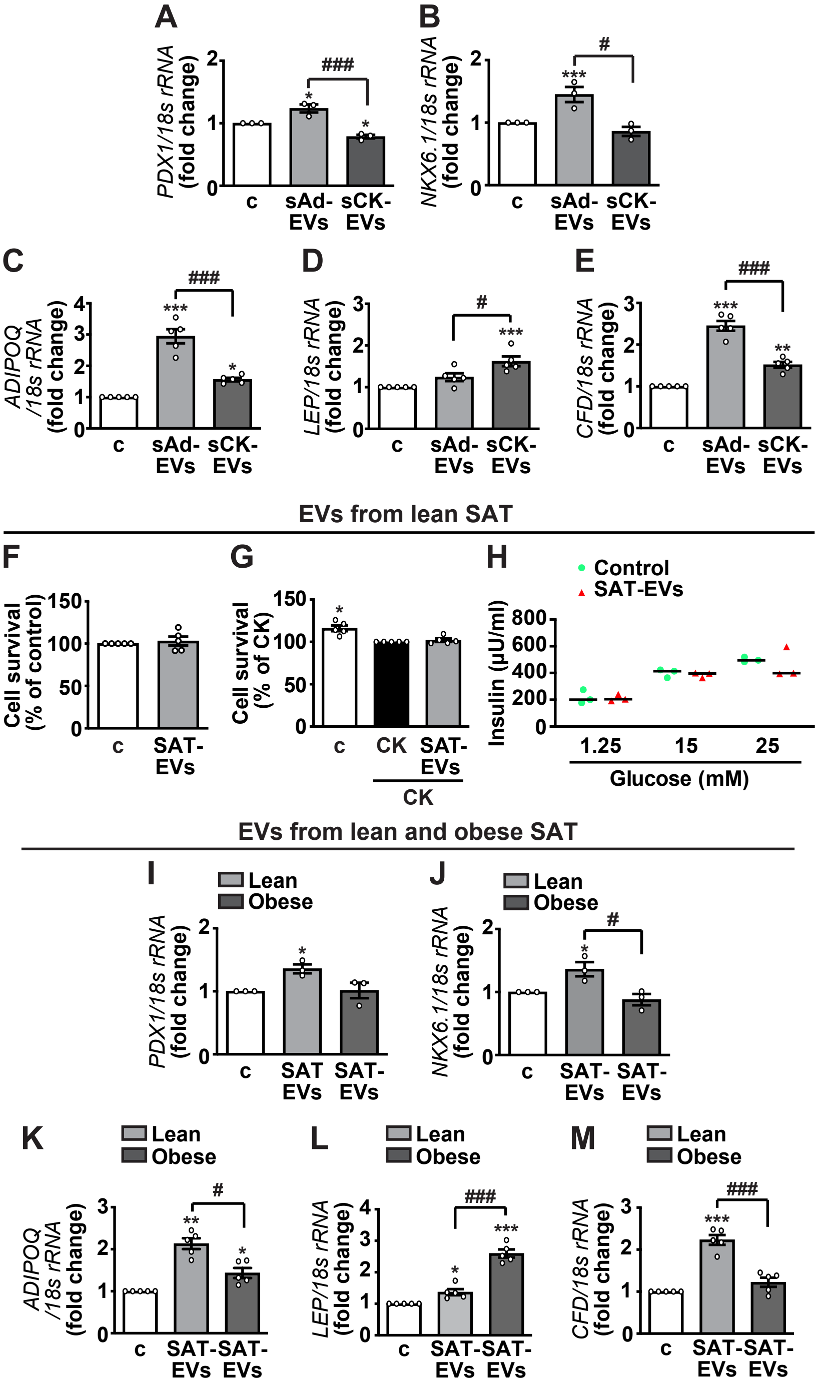

Figure S3. Effects of human lean and obese subcutaneous AT-derived EVs (SAT-EVs) in EndoC-βH3 cells. mRNA for Pdx1 **(A)**, Nkx6.1 **(B)**, adiponectin (*ADIPOQ*)= **(C)**, leptin (*LEP*) **(D)** and adipsin (*CFD*) **(E)**, assessed by real-time PCR in cells untreated (c, control) or treated with lean sAd-EVs or sCK-EVs for 24 h. Results are means ± SEM. $^{*}P < 0.05$, $^{**}P < 0.01$; $^{***}P < 0.001$; $^{\#}P < 0.05$, $^{\#\#}P < 0.01$ by ANOVA and Tukey's post-hoc test (n=3 for *PDX1* and *NKX6*.1; n=5 for *ADIPOQ*, *LEP* and *CFD*). **(F-G)** Survival (MTT) in cells cultured for 24 h in normal medium **(F)**, or in medium with TNF-α/IFN-γ/IL-1β (CK) (20, 20 and 1 ng/ml, respectively) **(G)**, and either without (c, control) or with lean SAT-EVs (10 x 10^3/cell). Results are means ± SEM. $^{*}P < 0.05$ vs. c by 2-tailed t test **(F)** or vs. CK by ANOVA and Tukey's post-hoc test **(G)** (n=5). **(H)** Insulin secretion in cells incubated with βKREBS®BSA buffer for 1 h, then for a further 1 h with the indicated concentrations of glucose, in either absence (Control) or presence of SAT-EVs. Results are means ± SEM. mRNA for Pdx1 **(I)**, Nkx6.1 **(J)**, adiponectin **(K)**, leptin **(L)** and adipsin **(M)** assessed by real-time PCR in β-cells untreated (c, control) or treated with lean or obese SAT-EVs for 24 h. Results are means ± SEM. $^{*}P < 0.05$, $^{**}P < 0.01$; $^{\#}P < 0.05$ by ANOVA and Tukey's post-hoc test (n=3 for *PDX1* and *NKX6*.1; n=5 for *ADIPOQ*, *LEP* and *CFD*).

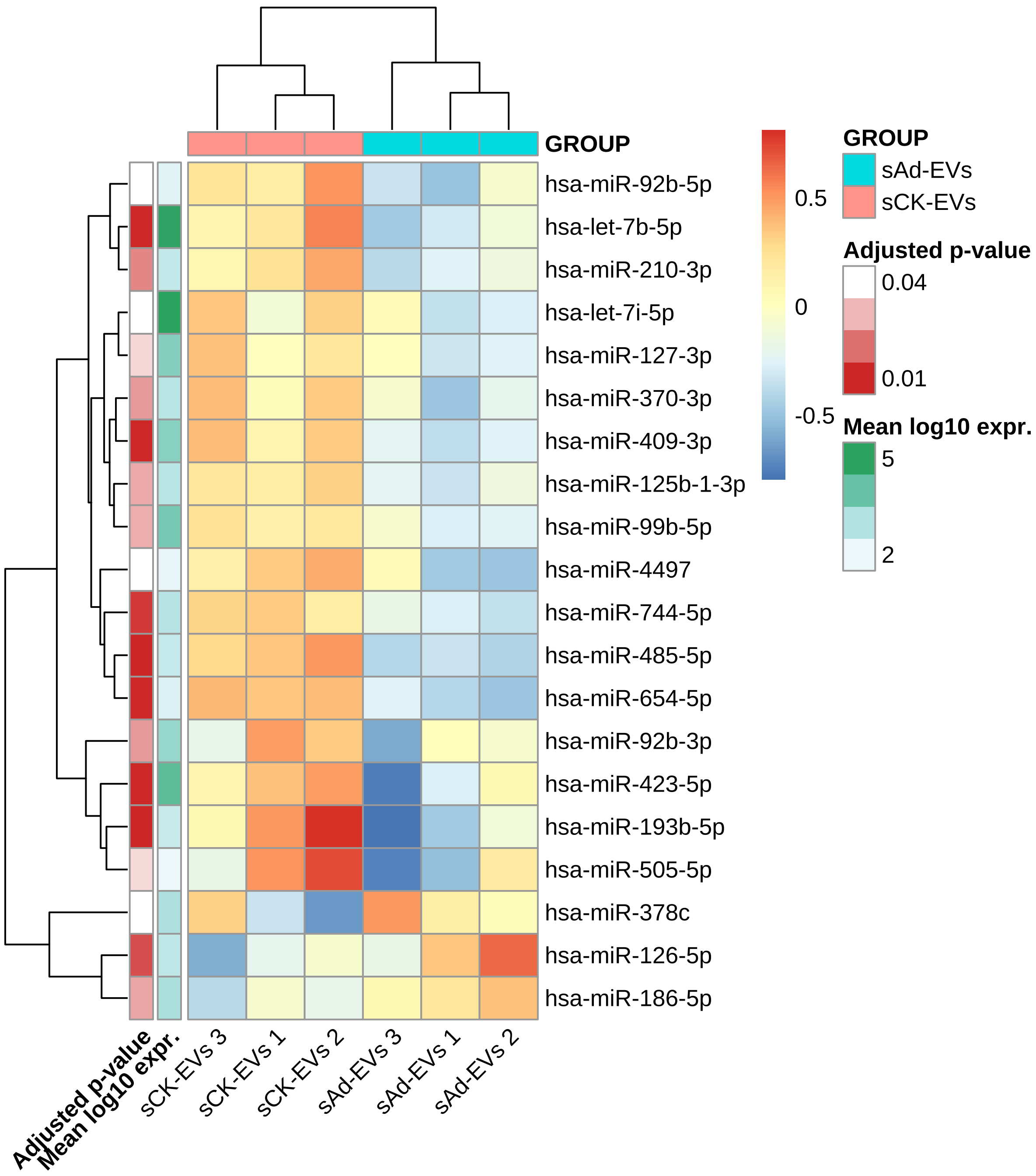

Figure S4. Figure S4. Heat map displaying the significantly differentially expressed miRNAs from small RNA-Seq in sAd-EVs and sCK-EVs. Each row represents a miRNA and each column a sample. The sample dendrogram, generated in an unsupervised way from the expression profiles, is shown at the top. The heat map colour shows the log2 fold change of each sample normalized expression with respect to the mean expression (across samples) for each miRNA: red, yellow and blue correspond to high, medium and low expression, respectively.

Table S1. Information on donors and human pancreatic islets.

	Gender	Age	BMI	Islets	
				Purity (%)	Viability (%)
Donor #1	Male	52	24.5	70	95
Donor #2	Female	48	25.4	60	90
Donor #3	Male	47	37.1	70	95
Donor #4	Male	52	26.2	80	90

BMI: body mass index

Table S2. Antibodies used in the study.

**Antibody**	**Source**	**Identifier**
Mouse monoclonal anti-Alix	Santa Cruz Biotechnology	Cat#sc-53540; RRID: AB_673819
Mouse monoclonal anti-PPARγ	Santa Cruz Biotechnology	Cat#sc-7273; RRID: AB_628115
Mouse monoclonal anti-Actin	Santa Cruz Biotechnology	Cat#sc-376421; RRID: AB_11149557
Mouse monoclonal anti-Integrin β1	Santa Cruz Biotechnology	Cat#sc-374429; RRID: AB_11012020
Rabbit polyclonal anti-FABP4	Abcam	Cat#ab23693; RRID: AB_447614
Rabbit polyclonal anti-CD63	Abcam	Cat#ab216130
Rabbit monoclonal anti-CD9	Abcam	Cat#ab92726; RRID: AB_10561589
Rabbit polyclonal anti-leptin	Abcam	Cat#ab3583; RRID: AB_303928
HRP-goat anti-rabbit IgG	SouthernBiotech	Cat#4050-05; RRID: AB_2795955
HRP goat anti-mouse IgG	SouthernBiotech	Cat#1010-05; RRID: AB_2728714
Rabbit polyclonal anti-phospho-Akt (Ser473)	Cell Signaling Technology	Cat#9271; RRID: AB_329825
Rabbit polyclonal anti-Akt	Cell Signaling Technology	Cat#9272; RRID: AB_329827
Rabbit polyclonal anti-phospho-GSK-3β (Ser9)	Cell Signaling Technology	Cat#9336; RRID: AB_331405
Rabbit monoclonal anti-GSK-3β	Cell Signaling Technology	Cat#9315; RRID: AB_490890
Rabbit polyclonal anti-phospho-p44/42 MAPK (Erk1/2) (Thr202/Tyr204)	Cell Signaling Technology	Cat#9101; RRID: AB_331646
Rabbit polyclonal anti-p44/42 MAPK (Erk1/2)	Cell Signaling Technology	Cat#9102; RRID: AB_330744
Rabbit monoclonal anti-Adiponectin (C45B10)	Cell Signaling Technology	Cat#2789; RRID: AB_2221630
Rabbit monoclonal phospho-PERK (Thr980) (16F8)	Cell Signalling Technology	Cat#3179; RRID: AB_2095853
Rabbit monoclonal PERK (C33E10)	Cell Signalling Technology	Cat#3192; RRID: AB_2095847
Rabbit monoclonal phospho-SAPK/JNK (Thr183/Tyr185) (81E11)	Cell Signalling Technology	Cat#4668; RRID: AB_823588
Rabbit polyclonal SAPK/JNK Antibody	Cell Signalling Technology	Cat#9252; RRID: AB_2250373
Rabbit polyclonal phospho-eIF2α (Ser51)	Cell Signaling Technology	Cat#9721; RRID: AB_330951
Rabbit polyclonal eIF2α Antibody	Cell Signaling Technology	Cat#9722; RRID: AB_2230924
Goat anti-rabbit Alexa Fluor 488	Invitrogen	Cat#A-11008; RRID: AB_143165

Table S3. RT-PCR primer sequences.

Gene	Species	Sequence Forward/Reverse	Accession Number
Adipoq	Mouse	5’-TGTAGGATTGTCAGTGGATCTG-3’<br>5’-GCTCTTCAGTTGTAGTTACGTCAT-3’	NM_009605.5
Lep	Mouse	5’-TCAGGTCAAGGTCTGGTTCC-3’<br>5’-AGGACACCATCCAGGCTCTC-3’	NM_008493.3
Fabp4	Mouse	5’-ATGTGCAGAAGTGGGATGGA-3’<br>5’-TGCAAATTTCAGTCCAGGGC-3’	NM_024406.3
Pparg1	Mouse	5’-AGACCACTCGCATTCCTTTG-3'<br>5’-CCTGTTGTAGAGCTGGGTCTTT-3’	NM_138712.3
Pparg2	Mouse	5’-GCTGTTATGGGTGAAACTCTG-3’<br>5’-ATAAGGTGGAGATGCAGGTTC -3’	NM_011146.2
ADIPOQ	Human	5’-TGGAGTACAGTGACACGACC-3’<br>5’-GTGGTGGAATGCACCTGAAG-3’	NM_004797.4
LEP	Human	5’-ACCAAGGTCTTCAGCCATCA-3’<br>5’-CTTGTGTTGCTGGGAGTTCC-3’	NM_000230.3
FABP4	Human	5’-GAAGTAGGAGTGGGCTTTGC-3'<br>5’-CTTCGTCAAATTCCTGGCCC-3’	NM_001442.2
PPARG1	Human	5’- AAAGAAGCCGACACTAAACC-3'<br>5’- CTTCCATTACGGAGAGATCC-3’	NM_138712.5
PPARG2	Human	5’- GCGATTCCTTCACTGATAC-3’<br>5’- CTTCCATTACGGAGAGATCC-3’	NM_015869.5
18s rRNA	Mouse/Human	5’-CCCATGGATGAAGTCTACC-3’<br>5’-GTCCTCCTCCTTTTTCCAC-3’	NR_146144.1

Table S4. Real-time PCR primer sequences.

Gene	Species	Sequence Forward/Reverse	Accession Number
Pdx1	Rat	5'-GGTGCCAGAGTTCAGTGCT-3'<br>5'-GGCACTTCGTATGGGGAGAT-3'	NM_022852.3
Nkx6.1	Rat	5'-TCAGGTCAAGGTCTGGTTCC-3'<br>5'-GTTCTCCGAAGTCCCCTTGA-3'	NM_031737.1
Adipoq	Rat	5'-AGGCCGTTCTCTTCACCTAC-3'<br>5'- TTGTCCCCTTCCCCATACAC-3'	NM_144744.3
Lep	Rat	5'-TGGACCAGACTCTGGCAGTC-3'<br>5'-AGGACACCATCCAGGCTCTC-3'	XM_008762762.2
Cfd	Rat	5'-AATCATGGACCGGAACACCT -3'<br>5'- AGATCCCCACGTAACCACAG-3'	NM_001077642.1
Tnfa	Rat	5'-TGAACTTCGGGGTGATCG-3'<br>5'-GGGCTTGTCACTCGAGTTT-3'	XM_008772775.2
Ifng	Rat	5'-AGTCTGAAGAACTATTTTAACTCAAGTAGCAT-3'<br>5'-CTGGCTCTCAAGTATTTTCGTGTTAC-3'	NM_138880.2
Il1b	Rat	5'-CACCTCTAAGCAGAGCACAG-3'<br>5'-GGGTTCCATGGTGAAGTCAAC-3'	NM_031512.2
Chop	Rat	5'-AGAGTGGTCAGTGCGCAGC-3'<br>5'-CTCATTCTCCTGCTCCTTCTCC-3'	NM_001109986.1
PDX1	Human	5'- CCCATGGATGAAGTCTACC -3'<br>5'- GTCCTCCTCCTTTTTCCAC -3'	NM_000209.4
NKX6.1	Human	5'-CTGGAGAAGACTTTCGAACAA-3'<br>5'-AGAGGCTTATTGTAGTCGTCG-3'	NM_006168.2
ADIPOQ	Human	5'-TGGAGTACAGTGACACGACC-3'<br>5'-GTGGTGGAATGCACCTGAAG-3'	NM_004797.4
LEP	Human	5'-ACCAAGGTCTTCAGCCATCA-3'<br>5'-CTTGTGTTGCTGGGAGTTCC-3'	NM_000230.3
CFD	Human	5'-GCAACAAAGTCCCGAGCAA-3'<br>5'-GGCCTCCCCTTCTGCATATAG-3'	NM_001928.3
TNFA	Human	5'-TCTCGAACCCCGAGTGACA-3'<br>5'- GGCCCGGCGGTTCA-3'	NM_000594.4
IFNG	Human	5'-AGCGGATAATGGAACTCTTTTCTTAG-3'<br>5'-AAGTTTGAAGTAAAAGGAGACAATTTGG-3'	NM_000619.3
IL1B	Human	5'-CACGATGCACCTGTACGAATCA-3'<br>5'-GTTGCTCCATATCCTGTCCCT-3'	NM_000576.2
18s rRNA	Rat/Human	5'-CCCATTCGAACGTCTGCCCTATC-3'<br>5'-TGCTGCCTTCCTTGGATGTGGTA-3'	NR_146144.1

Table S5. miRNAs significantly upregulated and downregulated in 3T3-L1-derived CK-EVs vs. Ad-EVs.

	miRNA name	Fold-change (in Log2)		miRNA name	Fold-change (in Log2)
Upregulated	mmu-miR-155-5p	4.29	Downregulated	mmu-miR-423-5p	-4.46
	mmu-miR-199b-5p	3.09		mmu-miR-320-3p	-4.04
	mmu-miR-34c-5p	2.89		mmu-let-7a-5p	-2.75
	mmu-let-7g-5p	2.00		mmu-miR-676-3p	-2.65
	mmu-miR-26b-5p	2.82		mmu-let-7e-5p	-2.18
	mmu-miR-210-3p	2.15		mmu-miR-25-3p	-2.27
	mmu-miR-30a-5p	1.73		mmu-miR-501-3p	-2.02
	mmu-miR-652-3p	3.53		mmu-miR-125a-5p	-2.25
	mmu-miR-199a-5p	1.66		mmu-miR-351-5p	-1.89
	mmu-miR-143-3p	1.59		mmu-miR-296-3p	-1.85
	mmu-miR-30b-5p	2.31		mmu-miR-486a-5p	-1.76
	mmu-miR-30a-3p	1.40		mmu-miR-328-3p	-1.55
	mmu-miR-182-5p	1.22		mmu-miR-2137	-1.72
	mmu-miR-708-5p	2.19		mmu-miR-92a-3p	-1.51
	mmu-miR-23a-3p	1.48		mmu-miR-10b-5p	-1.56
	mmu-miR-193b-3p	1.47		mmu-let-7d-5p	-1.50
	mmu-miR-455-5p	1.66		mmu-miR-1198-5p	-2.10
	mmu-miR-199a-3p	1.40		mmu-miR-224-5p	-1.40
	mmu-miR-20a-5p	1.90		mmu-miR-151-3p	-1.60
	mmu-miR-186-5p	1.45		mmu-miR-1981-5p	-1.61
	mmu-miR-93-5p	1.39		mmu-miR-484	-1.48
	mmu-miR-378a-5p	1.78		mmu-miR-99b-3p	-1.55
	mmu-miR-471-5p	1.20		mmu-miR-423-3p	-1.55
	mmu-miR-148b-3p	1.04		mmu-miR-99b-5p	-1.26
	mmu-miR-16-5p	1.54		mmu-miR-503-3p	-1.64
	mmu-miR-7a-5p	1.26		mmu-miR-744-5p	-1.28
	mmu-miR-146a-5p	1.08			

Data are shown as log2 fold-change relative to miRNA expression in CK-EVs compared to Ad-EVs.

Table S6. KEGG pathways enriched for target genes of the differentially expressed miRNAs in CK-EVs vs. Ad-EVs.

Molecular pathways	Adjusted P-value (BH)
AGE-RAGE signaling pathway in diabetic complications (mmu04933)	< 0.00001
p53 signaling pathway (mmu04115)	< 0.00001
NOD-like receptor signaling pathway (mmu04621)	< 0.00001
MicroRNAs in cancer (mmu05206)	0.0007
Pathways in cancer (mmu05200)	0.0007
Signaling pathways regulating pluripotency of stem cells (mmu04550)	0.0011
Inflammatory bowel disease (IBD) (mmu05321)	0.0028
Autophagy (mmu04140)	0.0030
Toll-like receptor signaling pathway (mmu04620)	0.0031
RIG-I-like receptor signaling pathway (mmu04622)	0.0032
Pancreatic cancer (mmu05212)	0.0044
PI3K/Akt signaling pathway (mmu04151)	0.0066
IL-17 signaling pathway (mmu04657)	0.0078
Endocrine resistance (mmu01522)	0.0083
Focal adhesion (mmu04510)	0.0128
C-type lectin receptor signaling pathway (mmu04625)	0.0144
Rap1 signaling pathway (mmu04015)	0.0149
JAK/STAT signaling pathway (mmu04630)	0.0164
Regulation of actin cytoskeleton (mmu04810)	0.0164
Relaxin signaling pathway (mmu04926)	0.0224
Estrogen signaling pathway (mmu04915)	0.0232
Cellular senescence (mmu04218)	0.0240
Chemokine signaling pathway (mmu04062)	0.0306
Wnt signaling pathway (mmu04310)	0.0306

Table S7. Gene Ontology (GO) Biological Processes enriched for target genes of the differentially expressed miRNAs in 3T3-L1 derived CK-EVs vs. Ad-EVs.

Biological Processes	Adjusted P-value (BH)
Lipopolysaccharide-mediated signaling pathway (GO:0031663)	< 0.00001
Negative regulation of interleukin-2 production (GO:0032703)	< 0.00001
Response to hypoxia (GO:0001666)	< 0.00001
Negative regulation of interferon-gamma production (GO:0032689)	< 0.00001
Nucleotide-excision repair (GO:0006289)	< 0.00001
Cellular response to lipopolysaccharide (GO:0071222)	< 0.00001
Vascular endothelial growth factor receptor signaling pathway (GO:0048010)	< 0.00001
Positive regulation of angiogenesis (GO:0045766)	< 0.00001
Positive regulation of interleukin-6 production (GO:0032755)	< 0.00001
Negative regulation of canonical Wnt signaling pathway (GO:0090090)	< 0.00001
Negative regulation of necroptotic process (GO:0060546)	< 0.00001
Regulation of cell cycle (GO:0051726)	< 0.00001
Positive regulation of I-kB kinase/NF-kB signaling (GO:0043123)	0.0003
Response to lipopolysaccharide (GO:0032496)	0.0003
Cytokine production (GO:0001816)	0.0003
Positive regulation of interleukin-1 beta secretion (GO:0050718)	0.0005
Ceramide biosynthetic process (GO:0046513)	0.0005
Telomere maintenance (GO:0000723)	0.0008
Positive regulation of endothelial cell proliferation (GO:0001938)	0.0014
Extrinsic apoptotic signaling pathway (GO:0097191)	0.0017
Response to cytokines (GO:0034097)	0.0029
Positive regulation of tumor necrosis factor production (GO:0032760)	0.0032
Positive regulation of NF-kB transcription factor activity (GO:0051092)	0.0073
Positive regulation of JNK cascade (GO:0046330)	0.0082
Intracellular signal transduction (GO:0035556)	0.0349
Regulation of translation (GO:0006417)	0.0365
Regulation of gene expression (GO:0010468)	0.0420

Table S8. Validated human and murine miRNAs targeting genes altered in rodent and human β-cells after treatment with EVs.

	Target gene	miRNA	Ref Seq ID	Position of binding region	Validated on MIRTAR
**Human**	*TNFA*	hsa-miR-17-5p	NM_000594	3UTR	MIRT734730
		hsa-miR-24-3p	NM_000594	3UTR	MIRT733712
		hsa-miR-34a-5p	NM_000594	CDS	MIRT733989
		hsa-miR-452-5p	NM_000594	CDS	MIRT053456
	IFNG	hsa-miR-24-3p	NM_000619	3UTR	MIRT437970
		hsa-miR-409-3p	NM_000619	CDS	MIRT007285
	IL1B	hsa-miR-21-5p	NM_000576	3UTR	MIRT005951
		hsa-miR-24-3p	NM_000576	3UTR	MIRT733711
		hsa-miR-204-5p	NM_000576	CDS	MIRT005830
	ADIPOQ	hsa-miR-30c-2-3p	NM_001177800	3UTR	MIRT691109
		hsa-miR-214-5p	NM_001177800	CDS	MIRT691123
		hsa-miR-30b-3p	NM_001177800	3UTR	MIRT691106
		hsa-miR-149-3p	NM_001177800	3UTR	MIRT691119
		hsa-miR-30c-1-3p	NM_001177800	3UTR	MIRT691110
		hsa-miR-363-5p	NM_001177800	3UTR	MIRT691114
		hsa-miR-383-3p	NM_001177800	3UTR	MIRT691125
		hsa-miR-450a-1-3p	NM_001177800	3UTR	MIRT691100
		hsa-miR-887-5p	NM_001177800	CDS	MIRT691097
		hsa-miR-3122	NM_001177800	3UTR	MIRT691099
		hsa-miR-3165	NM_001177800	3UTR	MIRT691112
		hsa-miR-3689a-3p	NM_001177800	3UTR	MIRT691105
		hsa-miR-3689b-3p	NM_001177800	3UTR	MIRT691104
		hsa-miR-3913-5p	NM_001177800	3UTR	MIRT691098
		hsa-miR-3689c	NM_001177800	3UTR	MIRT691103
		hsa-miR-4728-5p	NM_001177800	3UTR	MIRT691118
		hsa-miR-6513-5p	NM_001177800	3UTR	MIRT691096
		hsa-miR-6514-3p	NM_001177800	3UTR	MIRT691111
		hsa-miR-6745	NM_001177800	3UTR	MIRT691113
		hsa-miR-6779-5p	NM_001177800	3UTR	MIRT691102
		hsa-miR-6780a-5p	NM_001177800	3UTR	MIRT691101
		hsa-miR-6785-5p	NM_001177800	3UTR	MIRT691117
		hsa-miR-6788-5p	NM_001177800	3UTR	MIRT691108
		hsa-miR-6799-5p	NM_001177800	3UTR	MIRT691115
		hsa-miR-6811-3p	NM_001177800	CDS	MIRT691122
		hsa-miR-6883-5p	NM_001177800	3UTR	MIRT691116
		hsa-miR-7106-5p	NM_001177800	5UTR	MIRT691120
		hsa-miR-1273h-5p	NM_001177800	3UTR	MIRT691107
**Mouse**	*Tnfa*	mmu-miR-296-3p	NM_001278601	CDS	MIRT437937
		mmu-miR-298-5p	NM_001278601	CDS	MIRT005496
		mmu-miR-351-5p	NM_001278601	3UTR	MIRT005493
	Ifng	mmu-miR-125a-5p	NM_008337	3UTR	MIRT735154
	Il1b	mmu-miR-122-5p	NM_008361	3UTR	MIRT005985

Table S9. miRNAs significantly upregulated and downregulated in human sCK-EVs vs. sAd-EVs.

	miRNA name	Fold-change (in Log2)		miRNA name	Fold-change (in Log2)
Upregulated	hsa-miR-193b-5p	1.39	Downregulated	hsa-miR-126-5p	-0.84
	hsa-miR-485-5p	1.13		hsa-miR-186-5p	-0.61
	hsa-miR-423-5p	0.88		hsa-miR-378c	-0.64
	hsa-miR-654-5p	1.27			
	hsa-let-7b-5p	0.83			
	hsa-miR-409-3p	0.78			
	hsa-miR-744-5p	0.78			
	hsa-miR-210-3p	0.76			
	hsa-miR-370-3p	0.74			
	hsa-miR-92b-3p	0.59			
	hsa-miR-125b-1-3p	0.67			
	hsa-miR-99b-5p	0.53			
	hsa-miR-127-3p	0.54			
	hsa-miR-505-5p	1.43			
	hsa-miR-92b-5p	1.04			
	hsa-miR-4497	1.17			
	hsa-let-7i-5p	0.57			

Data are reported as log2 fold-change relative to miRNA expression in human sCK-EVs vs. sAd-EVs.

Table S10. GO biological process enrichment analysis on target genes of differentially expressed miRNAs in human sCK-EVs vs. sAd-EVs.

GO Biological Processes	Adjusted P-value (BH)
Negative regulation of cell cycle process (GO:0010948)	0.017
Negative regulation of transcription, DNA-templated (GO:0045892)	0.018
Regulation of acyl-CoA biosynthetic process (GO:0050812)	0.039
Regulation of acetyl-CoA biosynthetic process from pyruvate (GO:0010510)	0.039
Positive regulation of stress-activated protein kinase signaling cascade (GO:0070304)	0.040
Positive regulation of protein phosphorylation (GO:0001934)	0.040
Intracellular steroid hormone receptor signaling pathway (GO:0030518)	0.040
Positive regulation of protein modification process (GO:0031401)	0.040
Negative regulation of transcription from RNA polymerase II promoter (GO:0000122)	0.040
Regulation of DNA endoreduplication (GO:0032875)	0.052

BH = Benjamini Hochberg correction for multiple tests.
